# Hypoxanthine is a metabolic biomarker for inducing GSDME-dependent pyroptosis of endothelial cells during ischemic stroke

**DOI:** 10.7150/thno.100090

**Published:** 2024-09-16

**Authors:** Jing Ye, Xinyuan Bi, Shiyu Deng, Xianghui Wang, Ze Liu, Qian Suo, Jiao Wu, Haoran Chen, Yong Wang, Kun Qian, Rubing Shi, Jing Zhao, Guo-Yuan Yang, Jian Ye, Yaohui Tang

**Affiliations:** 1 School of Biomedical Engineering, Shanghai Jiao Tong University, Shanghai, 200030, China.; 2 Institute of Medical Robotics, Shanghai Jiao Tong University, Shanghai, 200127, China.; 3 Department of Neurology, Zhongshan Hospital, Fudan University, Shanghai 200030, China.; 4 Department of Neurology, Minhang Hospital, Fudan University, Shanghai, 201102, China.; 5 Shanghai Key Laboratory of Gynecologic Oncology, Ren Ji Hospital, School of Medicine, Shanghai Jiao Tong University, Shanghai 200127, China.

**Keywords:** Endothelial cells, GSDME, Hypoxanthine, Metabolic biomarker, Pyroptosis

## Abstract

**Rationale:** Stroke induces metabolic changes in the body, and metabolites have become potential biomarkers for stroke. However, the specific metabolites involved in stroke and the mechanisms underlying brain injury during stroke remain unclear.

**Methods:** Surface-enhanced Raman spectroscopy (SERS) and liquid chromatography-mass spectrometry (LC‒MS) analysis of clinical serum samples from 69 controls and 51 ischemic stroke patients who underwent reperfusion within 24 hours were performed to identify differentially abundant metabolites. Mice were subjected to transient middle cerebral artery occlusion (tMCAO) and then intravenously injected with hypoxanthine. The infarct area was evaluated via tetrazolium chloride (TTC) staining, and behavior tests were conducted. Blood-brain barrier (BBB) leakage was assessed by Evans blue and IgG staining. Human blood vessel organoids were used to investigate the mechanism of hypoxanthine-induced pyroptosis of endothelial cells.

**Results:** SERS and LC‒MS revealed the metabolic profiles of serum from stroke patients and controls with high sensitivity, speed and accuracy. Hypoxanthine levels were significantly elevated in the acute stage of ischemic stroke in both patients and mice (p < 0.001 after Bonferroni correction). In addition, increasing hypoxanthine increased the infarct area and aggravated BBB leakage and neurobehavioral deficits in mice after ischemic stroke. Further mechanistic studies using endothelial cells, human blood vessel organoids, and stroke mice demonstrated that hypoxanthine-mediated gasdermin E (GSDME)-dependent pyroptosis of endothelial cells occurs through intracellular Ca^2+^ overload.

**Conclusion:** Our study identified hypoxanthine as an important metabolite that induces vascular injury and BBB disruption in stroke through triggering GSDME-dependent pyroptosis of endothelial cells.

## Introduction

Stroke is a leading cause of mortality and morbidity worldwide 1. Blood‒brain barrier (BBB) disruption is a critical pathophysiological hallmark of stroke, resulting in severe clinical consequences such as brain edema, hemorrhagic transformation, and exacerbated neuroinflammation and brain injury. Thus, the BBB is an important therapeutic target for preventing further brain injury in stroke patients. However, the mechanisms underlying stroke-induced BBB disruption are still unclear. Stroke triggers complex metabolic changes, and the circulating metabolome can sensitively reflect pathophysiological events in the brain 2. Metabolite levels may directly indicate metabolic disturbances caused by stroke or may be indirectly related to secondary responses such as inflammation, BBB disruption, and oxidative stress. Therefore, changes in metabolite levels after stroke may reveal the underlying pathophysiological mechanisms and offer insight into disease causes and prognosis 3, 4.

Metabolic dysregulation is associated with BBB disruption after stroke 5, 6. In recent years, numerous studies have identified metabolites in patients with cerebral ischemia and animal models that could serve as biomarkers for stroke diagnosis and outcome prediction 7. For example, disturbances in arginine-related metabolism 8, 9 and hypoxanthine-related metabolism have been reported after stroke, with some related metabolites being closely associated with the pathological changes associated with stroke 10-13. Understanding how alterations in metabolite levels mediate endothelial cell death and BBB disruption provides valuable insights into stroke pathology and aids in the development of therapeutic strategies to mitigate brain injury.

Pyroptosis is a form of programmed cell death that activates and amplifies the inflammatory response through the release of many cytokines. Gasdermins, which are pore-forming proteins, cause cell membrane permeabilization and inflammasome-mediated pyroptosis. Among gasdermins, gasdermin D (GSDMD) and gasdermin E (GSDME) are the most extensively studied 14-16. Caspase-mediated cleavage of these proteins results in the release of an N-terminal fragment (GSDMD‒N or GSDME‒N) and a C-terminal fragment. The GSDMD‒N and GSDME‒N fragments bind to acidic phospholipids, oligomerize in target cells to form membrane pores, and lead to the leakage of inflammatory cytokines and alarm signals 17, 18. GSDME is cleaved by caspase 3 19, whereas GSDMD is cleaved by caspase 1 or caspase 11 in mice (caspase 4 or caspase 5 in humans) 20-22. Research on the involvement of pyroptosis in neurological diseases is expanding 23-25. For example, HIV infection triggers the activation of both caspase 1 and caspase 3 in the brain, leading to GSDME-mediated neuronal pyroptosis 26. Feng Shao's group found that GSDMD activation in brain endothelial cells mediates BBB breakdown during stroke 27. However, the role of metabolites in mediating endothelial cell pyroptosis after stroke remains to be investigated.

In this work, we utilized surface-enhanced Raman spectroscopy (SERS) and liquid chromatography-mass spectrometry (LC‒MS) to obtain the metabolic profiles of serum samples from stroke patients and controls with high sensitivity, speed and accuracy. We further explored the underlying mechanism of hypoxanthine-induced pyroptosis of endothelial cells and BBB disruption in stroke.

## Methods

### Mouse model of middle cerebral artery occlusion (MCAO)

Animal studies were performed in accordance with the Animal Research: Reporting *in Vivo* Experiments: ARRIVE guidelines, and approved by the Institutional Animal Care and Use Committee (IACUC) of Shanghai Jiao Tong University. Adult C57BL/6J mice (8 weeks, n=50, male) were anesthetized with 1.5% isoflurane and 30%/70% O_2_/NO on the heating pad. The MCAO surgery was performed as described in our previous study 28. A 6-0 suture was inserted into the internal carotid artery through the external carotid artery to occlude the middle cerebral artery (MCA). After 90 min, the suture was withdrawn for reperfusion. Blood flow was evaluated by laser Doppler flowmetry (Moor Instruments, Devon, UK). Regional blood flow decreased by 80% compared to baseline after ischemia and recovered to 80% of baseline after reperfusion is considered as a successful model.

### Collection of clinical samples

The double-blinded, randomized trial design and patient eligibility criteria have been previously reported 29. Briefly, male and female participant aged 45-80 with ischemic stroke within 24 hours after arterial thrombectomy or tPA therapy were enrolled in the study, the stroke patients were confirmed by Computed Tomography (CT) and Magnetic Resonance Imaging (MRI). A total of 120 clinical samples (51 ischemic stroke samples and 69 control samples) were collected from the department of neurology, Minhang hospital, Fudan University (Table S2). The collection and usage of clinical samples have been approved by the ethics committee of Minhang hospital, Fudan University (2021-008-01K). Consent documents were obtained from participants in this study. The clinical data including age, gender, medical history and initial National Institutes of Health Stroke Scale (NIHSS) were collected. For the stroke group, there were 27 cases of large artery atherosclerosis, 15 cases of cardio embolic stroke and 9 cases of artery occlusion. For each participant, 3-5 ml fasting venous blood were withdrawn by experienced nurses within 24 hours after stroke. After the centrifugation of the blood at 3000 rpm for 10 min under ambient condition, about 400 μl serum was obtained and stored at -80 ℃. Before measurement, all samples were thawed on ice at 2 - 4 ℃. Repeated freeze-thaw processes and long-time exposure to room temperature and light should be avoid in order to prevent changes in the metabolite composition. Thereafter, each serum sample was filtered with a 3 kDa-cutoff filter (Amicon Ultra-4, PLBC Ultracel-3, Millipore, Burlington, MA) by ultracentrifugation at 10000 rpm for 20 min to remove the macromolecules and the metabolic substances were collected for SERS measurement.

### Materials and instrumentations for SERS measurements

Trisodium citrate (TSC, 98%), silver nitrate (AgNO_3_, AR, 99.8%) and hypoxanthine (99%) were obtained from Aladdin. Adenine (HPLC, ≥ 99.5%) was purchased from Macklin. All materials were used as received without further purification. Ultrapure water (18.2 MΩ) was used for all experiments.

A Zetasizer Nano ZSP (Malvern, UK) was used to characterize the hydrodynamic diameter of the Ag colloids (temperature: 25 ℃, dispersant: water, material: Ag, reflective index: 0.54). The absorbance spectrum was acquired on a UV1900 UV-Vis spectrophotometer. A JEM-2100F transmission electron microscope (JEOL, Tokyo, Japan) was applied to characterize the colloidal morphology operated at 200 kV.

### Synthesis of SERS colloids

The citrate-reduced Ag colloids were synthesized according to Lee and Meisel's method 30 with slight modifications. Briefly, 75 ml AgNO_3_ solution (0.13 mg ml^-1^) was brought to a boil under constant stirring. 2 ml of TSC solution (10.1 mg/ml) was added dropwise within 2 min. The mixture was kept boiling for another 1 h and then cooled to room temperature under stirring. The colloidal suspension was stored in 4 ℃ without light exposure. Before being used, the suspension was ultrasonicated for 1 min to minimize colloidal aggregation and precipitation during storage.

### Preparation of the pure metabolite solution

For the standard spectra of hypoxanthine and adenine, pure water solutions of adenine (10^-3^ M) and hypoxanthine (10^-3^ M) were prepared.

### SERS measurement

For SERS measurement, all samples were then mixed with the citrate-Ag colloids (1.1 nM) at a ratio of 1:1, followed by 10 sec ultrasonication to avoid sedimentation and 30 min incubation under ambient condition for full adsorption. The sample-colloids mixture was ultrasonicated for 5 sec to minimize colloidal precipitation and then 10 µl was injected into a quartz capillary (I.D.: 1 mm, O.D.: 2 mm). Pointwise scanning (step size: 10 µm) was applied along the capillary on a confocal Raman system (Horiba, XploRA INV). We applied a 638 nm laser (power: 12.67 mW), a 10× objective lens and 1 sec of acquisition time per spectrum. 200 spectra were acquired for every serum sample.

### SERS spectral analysis

#### Spectral preprocessing

Each serum SERS spectrum was preprocessed by spike removal and baseline correction with software Labspec 6 (Horiba Scientific). The spectrum was normalized with the peak intensity within the band from 200 to 270 cm^-1^ (Eq. 1).


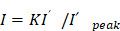
 (Equation 1)

(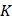: scaling factor, 1000 in this work; 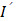: intensity before normalization)

#### Statistical and diagnostic methods

To estimate the number of spectra acquired for each serum sample, we computed the Pearson's correlation coefficient between the average spectra from two randomly selected subsets in the whole spectral set. Herein, for each subset size, the mean Pearson's correlation coefficient of the five computations with reassigned subsets was obtained.

For each sample, the mean spectrum was obtained by using the arithmetic mean at each wavenumber. Thereafter, the mean spectra corresponding to each serum sample were standardized. Sparse partial least squares discriminant analysis (sPLS-DA), performed on MetaboAnalyst 5.0, was used to reduce the dimension of the spectral data and achieve visualization in the 3-dimentional space. To build the diagnostic model for stroke, support vector machine (SVM) was applied with a linear kernel. 10-fold cross validation was used to optimize the model. The training set and the test set was reassigned 10 times with a ratio of 7:3. At each separation, the receiver operating characteristic (ROC) curve and the corresponding area under the curve (AUC) were computed to show the model performance.

To screen the biomarkers, Wilcoxon rank-sum test was applied to compare the intensities at each wavenumber from 200 to 2000 cm^-1^ (724 datapoints recorded for each spectrum by the instrument) between control and stroke group. The significantly varied spectral bands are identified when the p-value was smaller than 0.05 after Bonferroni correction.

### Liquid chromatography-mass spectrometry-based untargeted metabolomics

Serum was collected and stored at -80 °C, and thawed at 4 °C, equal volume from each sample was mixed for preparing quality control (QC), Adding 200 µl mix buffer (methyl alcohol and acetonitrile in 1:1 ratio, including 2 µg/ml chlorophenylalanine) into 50 µl serum, vortex and keep in -20 °C for over 2 h. Then centrifuge at 4 °C, 12000 rpm for 20 min, remove the supernatant and freeze-dry the supernatant. After that, the powder was dissolved in solution (methyl alcohol/water in 1:1 ratio volume), vortex and centrifuge at 4 °C, 12000 rpm for 20 min, then transfer the supernatant into sample tube for further testing. Negative and positive ion mode mass spectra were equipped within 0.1 ms over a mass full scan rage of 67-1000 amu. The capillary temperature is 320 °C. The spray voltage is 3.2 kV (positive mode) and 2.8 kV (negative mode).

Vanquish UHPLC system (consisting of a binary pump, a vacuum degasser, an autosampler and a column oven, Thermo fisher Scientific, Waltham, MA) and the UPLC condition conclude column ACCQUITY UPLC HSS T3 (100X 2.1 mm, particle size 1.7 µm, waters) were used. Equal volume of solvent A (water with 0.1% formic acid) and Solvent B (acetonitrile with 0.1% formic acid) were delivered to the chromatographic system at a flow rate of 0.4 ml/min. Data was first acquired in two ion modes (positive and negative) using the Xcalibur 3.0 software (Thermo fisher Scientific), and the results were then imported into Progenesis QI v 2.3 software (Waters, UK) for multivariate statistical analysis, including peak picking, alignment and normalization. Further statistical analysis was performed on Ezinfor V3.0.3 (Umetrics, Umea, Sweden).

According to the exact mass (Mass error < 5ppm), dd-MS, and comparison with the database HMDB5.0. Biomarkers are screened under the criteria of ANOVA *p* < 0.05, Fold change >1.5, OPLSDA model vip >1, Coefficient of variation CV% < 30%.

### Measurement of Intracellular Calcium Ion Concentration ([Ca^2+^]_i_)

The [Ca^2+^]_i_ concentration was measured with the Ca^2+^-sensitive fluorescent indicator, fluo-3AM, using a confocal laser scanning microscope (IX70 Fluoview; Olympus, Tokyo, Japan). bEnd.3 cells were seeded on a coverslip of 24-well plate for confocal laser scanning. The cells were pretreated with the reagents indicated for 24 h and then incubated with 5 μM fluo-3AM in a culture medium for 35 min at 37 °C. After washing three times with DMEM medium (FBS free), fluorescent images were scanned at an excitation wavelength of 488 nm using an argon laser and 530 nm longpass emission filter.

### Mitochondrial membrane potential measurement

JC-1 (5,5',6,6'-tetrachloro-1,1',3,3'-tetraethyl-benzimidazolylcarbocyanine iodide) fluorescent probe was used to examine mitochondrial membrane potential. In normal mitochondria, JC-1 aggregates in the mitochondrial matrix to form a polymer with strong red fluorescence emission (λ_ex_ = 585 nm, λ_em_ = 590 nm). When the mitochondrial membrane potential is low, JC-1 cannot aggregate in the matrix of mitochondria and produce green fluorescence (λ_ex_ = 514 nm, λ_em_ = 529 nm).

### Infarct volume assessment by Triphenyl Tetrazolium chloride (TTC) staining

Tetrazolium chloride (T-8877, Sigma-Aldrich, St. Louis, MO) was dissolved in PBS at 20 mg ml^-1^. Mice were euthanized and brains were cut into coronary slices (thickness: 2 mm). Fresh brain slices were stained with 2% TTC (vol vol^-1^) immediately for 30 min, and then fixed in 4% PFA. TTC labels non-injured tissue, leaving the infarct area white. Infarct area was calculated by subtracting the non-infarct area of the ipsilateral side from the area of the contralateral side using the ImageJ software (National Institutes of Health). Infarction area (%) = [Infarct area]/[Contralateral hemisphere area]X100. Infarct areas on each section were summed to give the total infarct area as 100%. We scan the brain section with ruler. N=5 mice per group.

### Evans Blue extravasation

The integrity of BBB was measured by Evans Blue (EB). Mice were injected with 2% EB (4 ml kg^-1^) via jugular vein, and euthanized and perfused with 20 ml ice PBS after 2 h circulation. The ipsilateral brain was dissected, weighed, and incubated with formamide solution. The supernatant was obtained after centrifugation at 12000 *g* for 10min. The quantity of EB extravasation was measured by spectrophotometer at a wavelength of 632 nm. The EB extravasation was quantified as previously reported 31.

### Reagent treatment

Intravenous injection of allopurinol (135mg.kg^-1^) or hypoxanthine (135 mg kg^-1^) was carried out from one day before tMCAO to the third day after tMCAO. Allopurinol or hypoxanthine (10 μg ml^-1^) was used to treat bEnd.3 cells and human blood vessel organoids.

### Immunostaining of human blood vessel organoids and bEnd.3 cells

Human blood vessel organoid samples were fixed in 4% paraformaldehyde (PFA, Sinopharm Chemical Reagent, China) for 5 min, and fully dehydrated in 30% sucrose for 1~2 days at 4°C until sink to the bottom, organoids cryosections (15 μm in thickness) and bEnd.3 cell glass coverslips were incubated with 0.3% Triton-100 (Sigma) and then blocked with 2% bovine serum albumin (BSA) (Thermo fisher Scientific), and incubated with rabbit anti mouse ZO-1 (1:200, 61-7300, Thermo fisher Scientific), goat anti mouse CD31 (1:200, AF3628, R&D, Minneapolis, MN), sheep anti human CD31 (1:400, AF806, R&D), rabbit anti human Cleaved-Caspase 3 (1:100, R23727, ZEN-BIOSCIENCE, Chengdu, China) at 4°C overnight. After rinsing with PBS for three times, the brain sections were incubated with the secondary antibodies including Alexa Fluor 488-conjugated donkey anti-rabbit (1:1000, A21206, Thermo fisher Scientific), Alexa Fluor 488-conjugated donkey anti-goat (1:1000, A11055, Thermo fisher Scientific), Alexa Fluor 488-conjugated donkey anti-sheep (1:1000, A11015, Thermo fisher Scientific), or Alexa Fluor 594-conjugated donkey anti-rabbit (1:1000, A21207, Thermo fisher Scientific), and one step TUNEL apoptosis assay kit for 2 h at room temperature. Then the slides were rinsed with PBS for three times, covered and sealed with antifade mounting medium with DAPI. Images were acquired by confocal microscope (Leica, Wetzlar, Germany).

### IgG staining

For IgG staining, a series of 20 μm in thickness and 200 μm in interval brain cryosections from the front of ischemic tissue to the end were collected. IgG staining was performed using Histostain^TM^ Plus Kit. Briefly, brain slices were incubated in 3% H_2_O_2_ for 20 min and blocked using goat serum for 20 min. Followed by incubating with biotinylated goat anti-mouse IgG solution (1:50, SP-0022, Bioss antibodies, Woburn, MA) for 20 min. DAB staining was used for the visualization of IgG signal (Vector Labs, Newark, CA), and the sections were counterstained with hematoxylin. The average IgG positive area was calculated using ImageJ software.

### Neurobehavioral tests

Behavior tests were performed at 1, 3, 7, and 14 days after tMCAO by an experimenter and recorder blinded to the experimental design.

(1) Modified neurologic severity score (mNSS): The mNSS includes a composite of motor, reflex, and balance tests. It is graded from 0 to 14, in which 0 represents the normal, and 14 indicates the most severe injury.

(2) Hanging wire test: This is to monitor muscle strength and condition over time. The mouse was first put in the midpoint of a metallic wire (55 cm long, 2 mm thick, 35 cm above the layer) with its fore limbs attached. If the mouse reached either side of the wire, the score was added by one; if mouse falls, the score was decreased by one. The test lasted for 3 min before the total score was recorded.

(3) Grid-walking test: The elevated grid-walking apparatus was manufactured using wire mesh with a grid area of 32/20/50 cm (length/width/height). Each mouse was placed at the grid center in the beginning and allowed to walk freely for 5 min. A camera was placed beneath the apparatus that allowed recording the animal's walking trace. A step was considered a stepping error (foot fault) if the foot went through the grid hole without adequate support. During this 5-min period, the total number of foot faults for each limb, along with the total number of steps, were counted. The foot fault index was calculated by [(contralateral faults-ipsilateral faults)/ total steps] × 100%.

### ESC culture

Human H9 embryonic stem cells (hESCs) were cultured on Geltrex (Thermo Fisher Scientific A1413302)-coated culture dishes in the mTeSR medium (Stemcell 85850, Vancouver, Canada). Cells were passaged every 5 days by digesting with ReleSR (Stemcell 100-0483) for 1 min at 37°C. Then, discard ReleSR and add 1 ml mTeSR medium at 37 °C for 6 min and transfer at 1:30 ratio onto fresh Geltrex-coated plates containing 2 ml mTeSR medium. On the following day, we replaced the medium with a new mTeSR medium every day.

### Generation of human blood vessel organoid

hESC9 clones were dissociated into single cells with Accutase (Stemcell 07920), then the cells were resuspended in the mTeSR1 medium (Stemcell 85850) containing 50 μM Y27632 (Stemcell 72308) and seeded into the V-bottom 96-well plate with 7000-9000 cells per aggregate, 150 μl per well to form embryonic bodies (EBs). On the next day, the culture medium was replaced by the mesodermal induction medium [APEL2 (Stemcell 05270) with 6 μM CHIR99021 (Stemcell 72054)]. On the fourth day, the mesodermal induction medium was replaced by the endothelial induction medium [(APEL2 with 50 ng/ml VEGF (91502ES10, YEASEN, Shanghai, China), 25 ng/ml BMP4 (92053ES10, YEASEN) and 10 ng/ml bFGF (91330ES10, YEASEN). On the seventh day, the medium was changed into the EGM medium (Lonza CC-3162, Hayward, CA) with 50 ng/ml VEGF for the maturation of endothelial cells (ECs), and the medium was changed every other day. From the twelfth day, the EBs were embedded into Matrigel droplets and cultured with the EGM medium with 20 ng/ml VEGF. The mature human blood vessel organoid was obtained after 28 days of culture.

### qRT-PCR

TRI reagent (TR118, MRC, Cincinnati, OH) was used to extract RNA from cortex and striatum of lesion area and surrounding area on tMCAO mouse brain at 3 days. The RNA was reverse transcribed using the Hifair II 1^st^ Strand cDNA Synthesis SuperMix for qPCR (gDNA digester plus) (11123ES60, YEASEN) following to the manufacture's protocol. Real-time fluorescent quantitative PCR was performed using Hieff qPCR SYBR Green master mix (High rox) (11203ES08, YEASEN) and recorded by 7900HT Fast Real-Time PCR System. Primers sequence for the targeted genes were provided in Table **S1**. The specific mRNA expression was analyzed by using the 2^-∆∆CT^ method and normalized to vehicle buffer treated tMCAO group.

### Western blot analysis

The lesion area of brain was harvested and lysed in RIPA lysis buffer (20-188, EMD Millipore, Burlington, MA) in the presence of a protease inhibitor cocktail and phosphatase inhibitor cocktail (catalog number:11697498001, ROCHE, Basel, Switzerland), brain tissues were homogenized and centrifuged at 15,000 *g* for 20 min at 4°C after incubated at ice 30 min. The supernatant proteins concentration were quantified by BCA Protein Assay Kit, separated running by SDS-PAGE, and incubated in polyvinylidene fluoride membranes (Millipore) with specific primary antibodies 4°C overnight, then incubated with a specific horseradish peroxidase (HRP)-conjugated secondary antibody at room temperature for 1 h. The relative intensities of the stripe were determined by the Image J software. See Table S3 for the detailed antibodies.

### Data analysis and statistics for mice, cells and organoids experiments

The related data were tested for normal distribution (Shapiro-Wilk test) and equality of variances using GraphPad Prism (version 8.0 for mac, GraphPad Software, Boston, MA, www.graphpad.com). A two-tailed student's t test was used for the pairwise comparison between two groups or Mann-whitney test. For normally distributed variables, comparison was examined with one-way or two-way analysis of variance, followed by Holm-Sidak correction and the data are presented as mean ± standard deviation (SD). Otherwise, if data does not comply with the normal distribution, the data were analyzed by Kruskal-Wallis test, with Dunnett's correction. The number of organiods and animals used (*n*) is indicated in the figure legends. Results with P < 0.05 were considered statistically significant.

## Results

### Identification of metabolic changes in ischemic stroke via SERS

**Figure 1:**
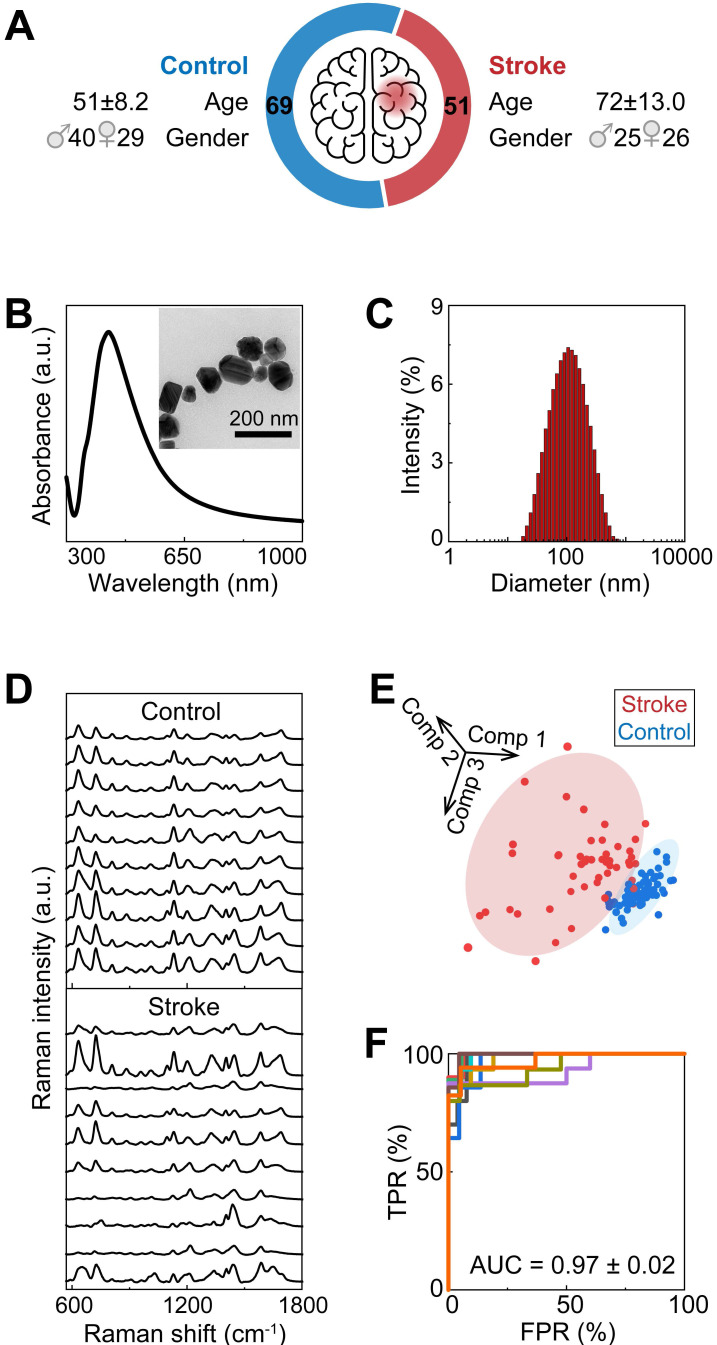
** SERS analysis of human serum for rapid metabolic profiling and diagnosis.** (**A**) A brief summary of the demographic information of the healthy control and stroke participants. (**B**) The absorbance spectrum (inset: TEM image) and (**C**) the hydrodynamic diameter of cit-Ag colloids. (**D**) Typical mean spectra of control and stroke samples. (**E**) Visualization of the mean spectra via sPLS-DA. (**F**) ROC curves of SVM-LDA from 10 repeated computations with the mean spectra on the reassigned training and test sets. The mean AUC was 0.97 ± 0.02.

To elucidate the metabolic changes associated with ischemic stroke, we collected serum samples from 51 ischemic stroke patients and 69 control participants (**Figure 1A**) with approval by the Institutional Review Board (IRB) of our hospital (see Methods). For the discover of biomarkers in human serum, nontargeting metabolic detection techniques with high multiplexity and sensitivity are preferred. SERS, which reflects the vibrational fingerprints of molecular species, was thus applied in this work given its ultrahigh sensitivity to the single-molecule level in colloidal electromagnetic (EM) hotspots and favorable tractability in both pretreatment and measurement. We used citrate-reduced silver (cit-Ag) colloids, which are stable during both storage and measurement in biosample mixtures 32, as the SERS substrate. They exhibited a resonance peak at 425 nm (**Figure 1B**) and a hydrodynamic diameter of 133.7 ± 5.13 nm (n = 3) (**Figure 1C**), which was consistent with the transmission electron microscopy results (**Figure 1B**). The serum samples were filtered with a 3 kDa cutoff in advance to prevent macromolecules (such as proteins) from occupying the EM hotspots and interfering with the detection of metabolites and then mixed with the cit-Ag colloids for measurement. Since the stochasticity of the molecules and the EM hotspots may cause fluctuations among spectra collected from different locations in the sample-colloid suspension (**Figure S1a**), the mean of 200 spectra was used for reproducible profiling 33. The number of spectra (i.e., 200) was determined by Pearson's correlation coefficient (**Figure S1b**) 34. The control group exhibited a more consistent spectral pattern, while there was relatively greater heterogeneity among stroke samples (**Figure 1D**), which was probably due to differences in ischemic stroke subtype resulting from variations in lesion location and severity 35, 36. The distribution of metabolic profiles was visualized primarily via a dimension reduction algorithm, i.e., through sparse partial least squares-discriminant analysis (sPLS-DA). As expected, the two groups showed distinctive distributions, with an error rate as low as 9.8% when 7 components (5-fold cross validation) were used. In addition, a more concentrated distribution was observed in the control group, while the distribution was more diffuse in the stroke group (**Figure 1E**). Discrimination between the two groups was further achieved by establishing a support vector machine (SVM) model with 70% of the samples as the training set and 30% as the test set. The SVM was trained 10 times on randomly reassigned training and test sets, showing robust differentiation between the two groups, with an average area under the receiver operating characteristic curve (AUC) of 0.97 (standard deviation = 0.02, **Figure 1F**), elucidating the potential of using SERS-derived serum metabolic profiles for accurate diagnosis of stroke.

**Figure 2:**
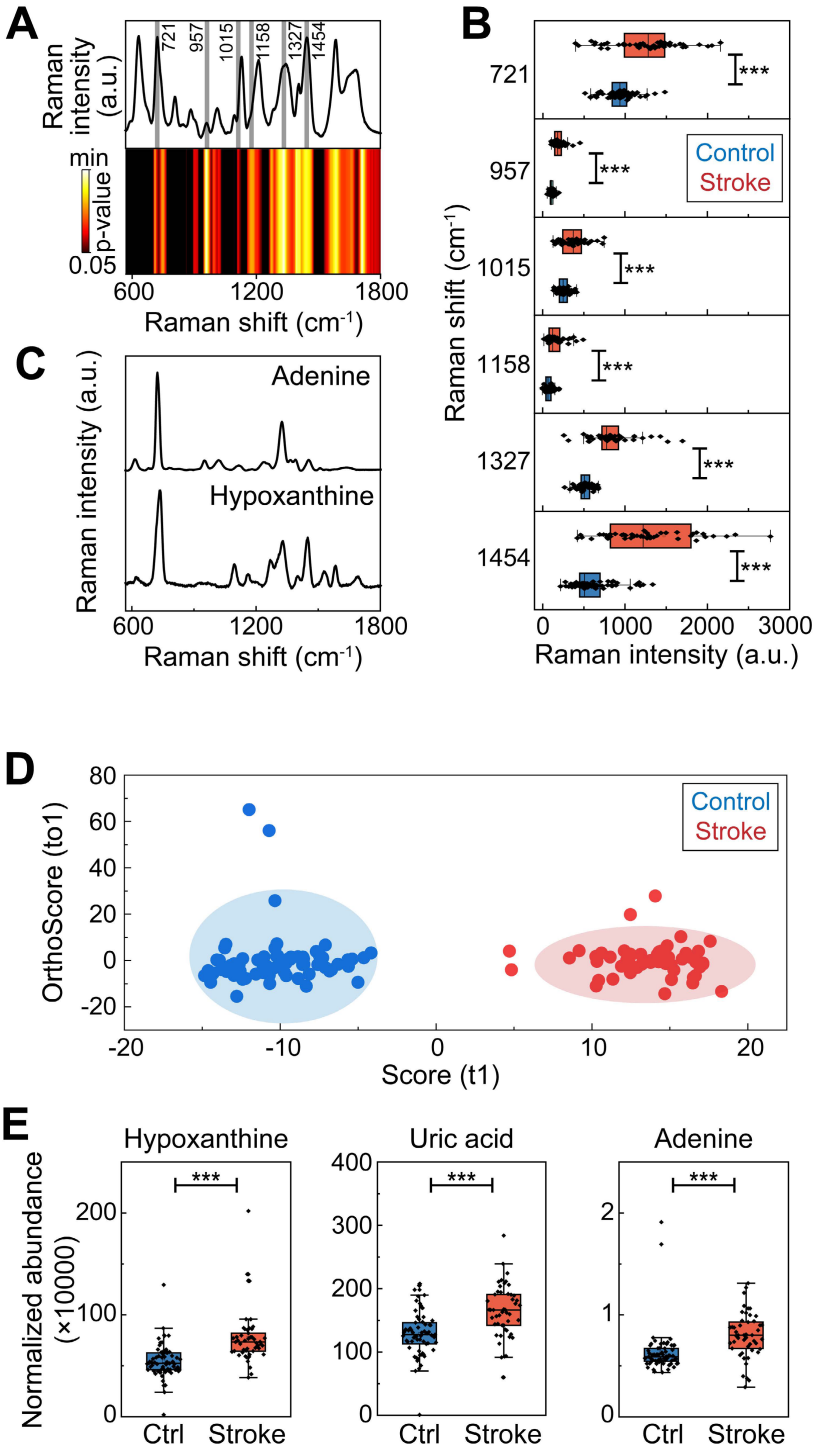
** Biomarker screening for ischemic stroke.** (**A**) The mean SERS spectra of all the serum samples and a heatmap of the Wilcoxon rank-sum test p values obtained by comparing the stroke group and the control group after Bonferroni's correction. (**B**) Typical spectral bands screened according to *p*< 0.05 by the Wilcoxon rank-sum test after Bonferroni's correction (also labeled by the gray stripes in panel (A)). N = 69 for the control group, N = 51 for the stroke group. *: *p* < 0.05, **: *p* < 0.01, ***: *p* < 0.001, vs. the control group. (**C**) Standard SERS spectra of adenine and hypoxanthine. (**D**) OPLS-DA score plots of the control patients and ischemic stroke patients according to HPLC‒MS-MS in positive ion mode. (**E**) Box plots of the normalized abundance of hypoxanthine, uric acid and adenine (ANOVA, *p* < 0.05, fold change > 1.3, OPSLSDA mode VIP > 1, coefficient of variation (CV)% < 30%, screened by Progenesis MetaScope, HMDB 5.0, serum, HMDB 2021 MetaScope search parameter database). N = 51 for the stroke group. ***: *p* < 0.001, vs. the control group.

To identify potential biomarkers, we applied the Wilcoxon rank-sum test to screen the spectral signatures potentially indicating differentially abundant metabolites in the serum of stroke patients (**Figure 2A**). As expected, the various spectral bands primarily matched some of the signatures in the serum spectra, and the most significantly varied bands (p < 0.05 after Bonferroni correction) were present at approximately the peak positions (**Figure 2B**). According to previous studies, these peaks coincide with the SERS fingerprints of hypoxanthine and adenine (**Figure 2C**), which are relatively highly abundant in human serum and have sufficient SERS detectability when our method is used at physiological levels 37, 38 (**Figure S2**). In this study, adenine exhibited SERS peaks at 721 (in-plane ring breathing), 957 (in-plane five-membered ring and six-membered ring deformation), 1015 (in-plane NH_2_ rocking) and 1327 cm^-1^ (in-plane C‒N stretching, C‒C stretching and N‒H bending) 39. Hypoxanthine showed SERS peaks at 721 (purine ring breathing), 1158 (O-H/C-H rocking), 1327 (C-H/C-H rocking) and 1454 cm^-1^ (imidazole ring stretching) 40. To further confirm these results, conventional LC‒MS was performed on identical serum samples, and the results were consistent, revealing differences in metabolomic profiles between the ischemic stroke group and the control group, as shown by orthogonal partial least squares-discriminant analysis (OPLS-DA) (**Figure 2D, S3**) (**Table S4**). Despite the different selection rules between MS and SERS caused by both pretreatment and measurement, hypoxanthine and adenine were consistently found to be upregulated in ischemic stroke by MS, verifying that these metabolites are potential biomarkers for stroke. In addition, uric acid was also found by MS to be dysregulated in ischemic stroke (**Figure 2E, S4**).

### Activation of the hypoxanthine metabolism pathway in ischemic stroke

**Figure 3:**
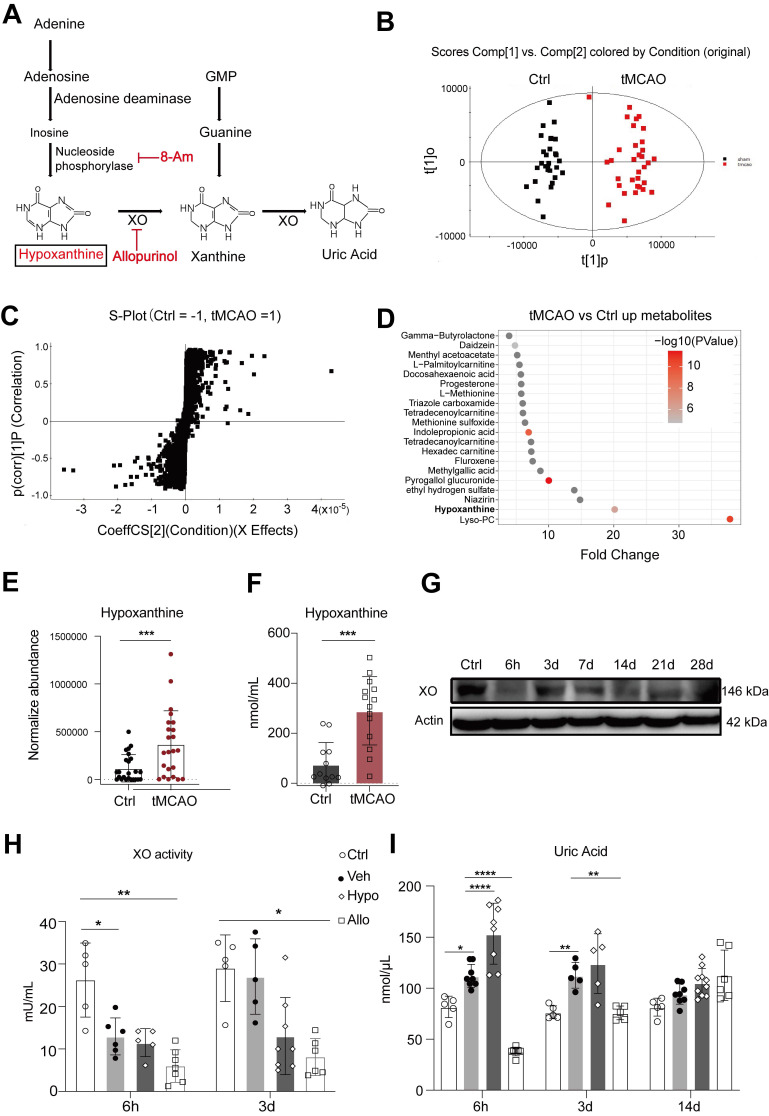
** Hypoxanthine was upregulated in ischemic stroke mice.** (**A**) Schematic diagram showing the adenine-hypoxanthine-uric acid catabolism pathway. Hypoxanthine can be converted to xanthine by XO and ultimately to uric acid. 8-Aminoinosine (8-Am) is an inhibitor of nucleoside phosphorylase, which leads to decreased hypoxanthine production. Allopurinol is an inhibitor of XO, which leads to decreased uric acid production and hypoxanthine accumulation. (**B**) OPLS-DA score plots for the control (sham) mice and tMCAO mice according to HPLC‒MS-MS in positive ion mode. (**C**) S-plot for tMCAO mice in positive mode. (**D**) Dot plot of the top 20 upregulated metabolites of tMCAO mice serum compare to control group. (**E**) Box plots showing the normalized abundance of hypoxanthine (ANOVA, *p* < 0.05, fold change >1.3, OPSLSDA mode VIP >1, coefficient of variation (CV) <3 0%, screened by Progenesis MetaScope, HMDB 5.0, serum, HMDB 2021 MetaScope search parameter database). N = 33 for the tMCAO group, N=27 for the control (sham) group. (**F**) Hypoxanthine/xanthine assays were used to measure the concentration of hypoxanthine in the serum of sham mice and tMCAO mice. (**G**) Representative Western blot image of XO expression in the brains of tMCAO and sham mice at different time points after stroke. (**H, I**) Quantitative analysis of XO activity and the concentration of uric acid in the serum of stroke mice treated with vehicle, hypoxanthine or allopurinol. n=5-6 per group. **E, F** Mann‒Whitney test (nonparametric test), H, **I** Two-way ANOVA with Tukey's multiple comparisons test. The data are the mean ± SD. *: *p* < 0.05, **: *p* < 0.01, ***: *p* < 0.001, vs. the control group.

The changes in adenosine, hypoxanthine, and uric acid levels after stroke indicate that the purine catabolism pathway, a purine salvage pathway, is involved in the pathophysiology of ischemic stroke in the acute stage. Hypoxanthine, an adenine intermediate, is converted to xanthine by xanthine oxidase (XO) and ultimately to uric acid (**Figure 3A**). In particular, both SERS and LC‒MS confirmed that hypoxanthine was upregulated in the ischemic stroke group, and a previous study suggested its relationship with brain edema 41. Thus, we investigated the function and metabolism of hypoxanthine in stroke.

A 90-min transient middle cerebral artery occlusion (tMCAO) mouse model was generated, and LC‒MS was performed on mouse serum to confirm whether hypoxanthine metabolism was increased in ischemic stroke mice. The differences in metabolomic profiles between the tMCAO group and the control group are shown by OPLS-DA (**Figure 3B**) (**Table S5**). There were numerous differentially expressed metabolites between tMCAO mice and sham mice, including LysoPC, Niazirin, indolepropionic acid, methionine (**Figure 3C, 3D**), which were similar to those between ischemic patients and controls. In particular, hypoxanthine was upregulated in stroke mice (**Figure 3E**), and this finding was validated by probe measurements in mice at 6 hours after tMCAO (**Figure 3F**). Since hypoxanthine is well documented to be converted to xanthine by XO, we also tested XO expression levels in the serum of mice at different timepoints after ischemic stroke via Western blotting. XO expression decreased at 6 h after tMCAO and gradually increased from day 3 to day 28 (**Figure 3G**). In addition, the activity of XO was lower at 6 hours after ischemic stroke than in the control group (sham mice) (**Figure 3H**), suggesting that the increase in hypoxanthine expression at 6 hours after stroke was due to failed conversion of hypoxanthine to uric acid. The level of the final catabolism product, uric acid, was increased in hypoxanthine-injected tMCAO mice compared with vehicle-treated tMCAO mice (**Figure 3I**). In addition, injection of allopurinol, an XO inhibitor, decreased uric acid levels, which may have been due to the inhibition of hypoxanthine conversion to uric acid, resulting in hypoxanthine accumulation.

### Hypoxanthine aggravated BBB leakage and neurobehavioral deficits in ischemic stroke mice

**Figure 4:**
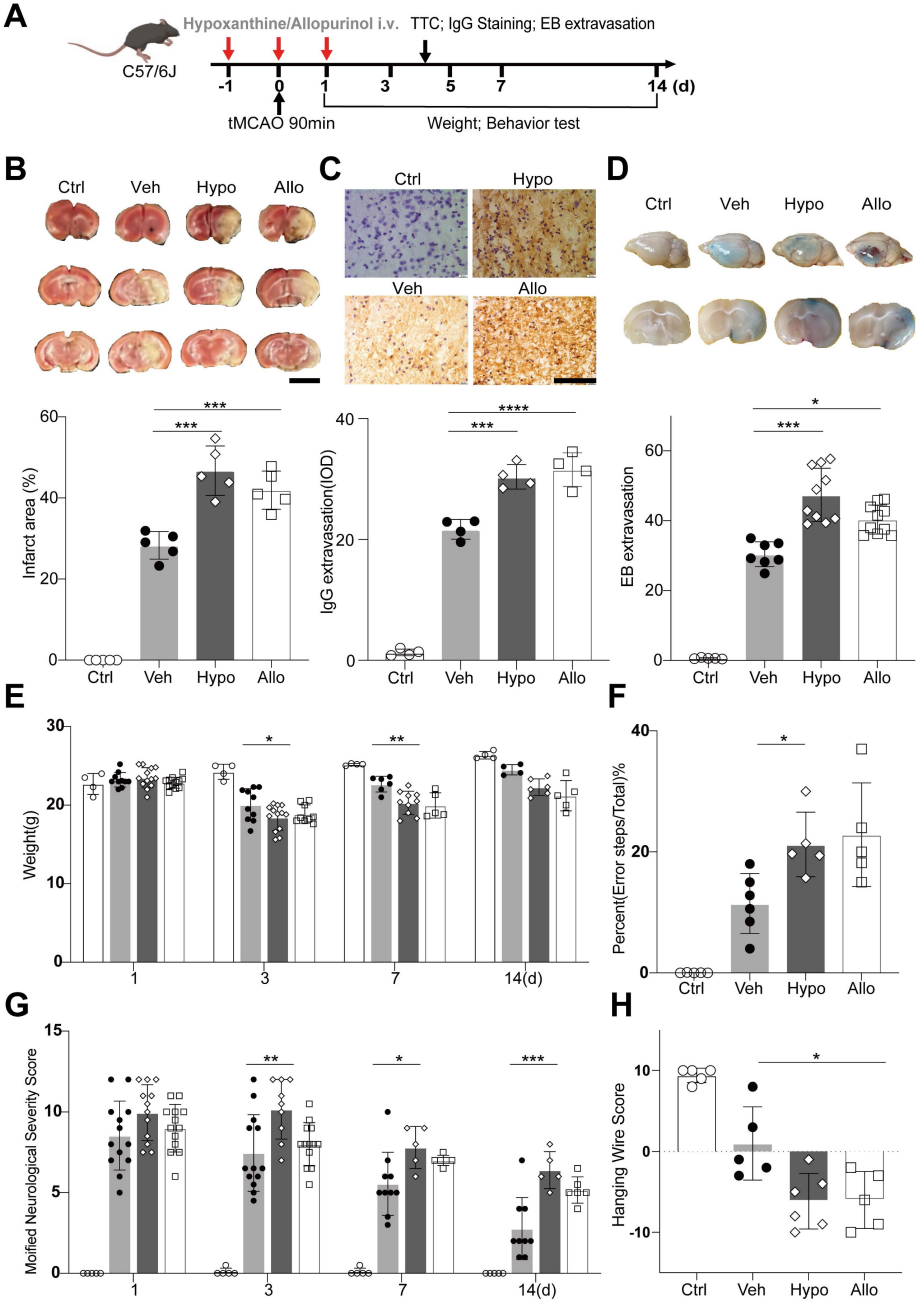
** Hypoxanthine aggravated BBB leakage and neurobehavioral deficits after ischemic stroke.** (**A**) Schematic of the experimental design. (**B**) TTC staining showing the infarct area of stroke mice treated with vehicle (Veh), hypoxanthine (Hypo) or allopurinol (Allo). N = 5 per group. Ctrl indicates sham mice. Scale bar: 5mm. (**C**) Photographs showing IgG staining in the ipsilateral lesional area in stroke mice treated with vehicle, hypoxanthine or allopurinol or the corresponding region in the control group (sham mice). N= 4 per group. Scale bar: 100 μm. (**D**) Representative Evans blue staining image of perfused whole brains (upper) and 2 mm-thick brain coronal slices (lower) from stroke mice treated with vehicle, hypoxanthine or allopurinol. N= 5~10 per group. Body weight (**E**) at different timepoints, performance in the grid walking test (**F**), the mNSS (**G**) and performance in the hanging wire test (**H**) were assessed for control (sham) mice and stroke mice treated with vehicle, hypoxanthine or allopurinol. N= 4 per group. **B**, **C**, **D**, **F**, **H** One-way ANOVA with Dunnett's multiple comparisons test. **E**, Two-way ANOVA with the Bonferroni's multiple comparisons test. **G** Two-way ANOVA with Tukey's multiple comparisons test. The data are the mean ± SD. *: *p* < 0.05, **: *p* < 0.01, ***: *p* < 0.001, ****: *p* < 0.0001, vs. the control group.

We then examined the effects of hypoxanthine on BBB integrity and neurological function in mice after ischemic stroke. Hypoxanthine or allopurinol was injected into mice from one day before tMCAO to one day after tMCAO (**Figure 4A**). Allopurinol, an XO inhibitor, can inhibit the conversion of hypoxanthine to xanthine and xanthine to uric acid, leading to the accumulation of hypoxanthine in mice. We found that hypoxanthine treatment and allopurinol treatment consistently increased the brain infarct volume (**Figure 4B**) and aggravated BBB leakage, as shown by IgG staining (**Figure 4C**) and Evans blue extravasation assay (**Figure 4D**).

To determine whether hypoxanthine impairs the neurobehavioral function of mice after stroke, we performed neurobehavioral tests, namely, the modified neurological severity score (mNSS) test, grid walking test, hanging wire test and body weight test, on days 1, 3, 5, 7 and 14. The results revealed that the mNSS in the hypoxanthine group and allopurinol group was greater than that in the vehicle group (tMCAO + vehicle) and control group (sham mice) (**Figure 4G**), suggesting more severe neurobehavioral deficits. Hypoxanthine- and allopurinol-treated mice performed worse in the grid walking test (**Figure 4F**) and hanging wire test (**Figure 4H**). The body weights of the hypoxanthine- and allopurinol-treated mice were lower than those of the control mice (**Figure 4E**). These data suggest that hypoxanthine plays a detrimental role in ischemic stroke.

### Hypoxanthine caused endothelial cell death and intracellular Ca2+ overload

**Figure 5:**
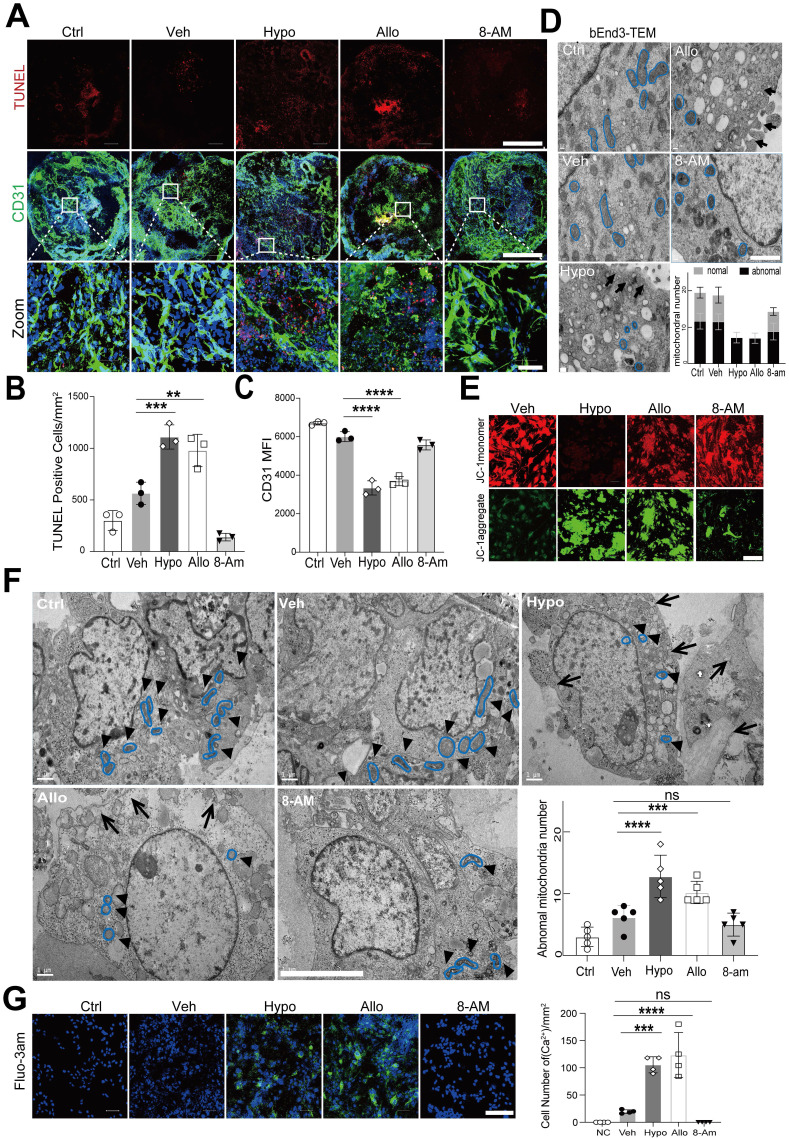
** Hypoxanthine induced endothelial cell death, mitochondrial dysfunction and intracellular Ca^2+^ accumulation.** (**A**) Immunostaining revealed CD31^+^ (green) and TUNEL^+^ (red) cells in human blood vessel organoids treated with vehicle, hypoxanthine, allopurinol or 8-minoinosine at 24 h after oxygen-glucose deprivation (OGD). Human blood vessel organoids without OGD treatment were defined as the control group (Ctrl). Scale bar: top and middle, 500 µm; bottom, 50 µm. (**B, C**) Bar graphs showing the number of TUNEL^+^ cells per mm^2^ and the mean fluorescence intensity (MFI) of the CD31^+^ signals. (**D**) TEM images showing the microstructure of endothelial cells treated with vehicle, hypoxanthine, allopurinol and 8-aminoinosine at 24 h after OGD. The mitochondrial microstructure is shown in blue, and the black arrows indicate swelling of the cell membrane and organelle cavitation. Semiquantification of the TEM data revealed the number of abnormal and normal mitochondria. N = 5 per group. Scale bar, 1 µm. (**E**) Representative images of the mitochondrial membrane potential of endothelial cells at 24 h after OGD. Scale bar, 100 µm. (**F**) TEM images of the endothelial microstructure of human blood vessel organoids treated with vehicle, hypoxanthine, allopurinol and 8-aminoinosine at 24 h after OGD. The bar graph shows the number of abnormal mitochondria in each cell; the black arrows show swelling of the cell membrane and organelle cavitation; the mitochondrial microstructures are indicated by blue borders; and the black triangles show changes in the mitochondrial microstructure, N = 5 per group. Scale bar, 5 µm. (**G**) Representative images of Fluo-3am fluorescence-labeled Ca^2+^ (green) in bEnd.3 cells treated with vehicle, hypoxanthine, allopurinol or 8-aminoinosine at 24 h after OGD. Scale bar, 100 µm. Bar graph showing the number of Ca^2+^-positive endothelial cells. N = 4 per group. **B**, **C**, **F**, **G** One-way ANOVA with Dunnett's multiple comparisons test. The data are the mean ± SD. *: *p* < 0.05, **: *p* < 0.01, ***: *p* < 0.001, ****: *p* < 0.0001, vs. the control group.

We then explored the effect of hypoxanthine on endothelial cells, which are the major components of the BBB. We observed that the level of the tight junction protein ZO1 was decreased in bEnd3 cells after hypoxanthine or allopurinol treatment (**Figure S5**). In addition, considering the limitations of cell experiments, we evaluated the effect of hypoxanthine on human blood vessel organoids. The data revealed that hypoxanthine or allopurinol treatment increased the number of TUNEL+ endothelial cells, reduced the number of CD31+ endothelial cells and disrupted vessel integrity (**Figure 5A-5C**).

TEM was further used to identify the effect of hypoxanthine on the ultrastructure of endothelial cells. The results revealed that the endothelial cells in the vehicle group had a normal cellular morphology, with an intact membrane and abundant normal mitochondria, whereas those in the hypoxanthine and allopurinol groups exhibited cytoplasmic edema, swelling and breakage of the cell membrane, and severe cavitation. Moreover, pyroptosis-like features, such as abnormal shrinkage of mitochondria, round mitochondria with altered nuclei, and cell membrane pores and swelling, were observed in bEnd.3 endothelial cells after hypoxanthine or allopurinol treatment (**Figure 5D**). A similar phenomenon was also observed in the human blood vessel organoids (**Figure 5F**). We then assessed the mitochondrial membrane potential via JC-1 staining. Normal cell mitochondria with a high membrane potential appeared orange, whereas mitochondria with a low membrane potential appeared green. We found that hypoxanthine or allopurinol treatment reduced the mitochondrial membrane potential (**Figure 5E**). In addition, by measuring intracellular Ca^2+^ concentrations in endothelial cells, we found that hypoxanthine or allopurinol treatment strongly increased the Ca^2+^ concentration in the cytoplasm of endothelial cells (**Figure 5G, S6**).

### Hypoxanthine induced GSDME-dependent pyroptosis of endothelial cells, which was mediated by Ca^2+^ overload

To investigate whether hypoxanthine-induced Ca^2+^ overload caused the death of endothelial cells, 1,2-bis(2-aminophenoxy) ethane-N,N,N',N'-tetra acetic acid (BAPTA-3am), a highly selective cytosolic calcium chelator, was used to chelate cytosolic Ca^2+^ (the 3am tag facilitated the intracellular delivery of BAPTA-3am). The results showed that BAPTA effectively reduced intracellular Ca^2+^ concentrations in endothelial cells in a dose-dependent manner (**Figure S6, 6A**).

**Figure 6:**
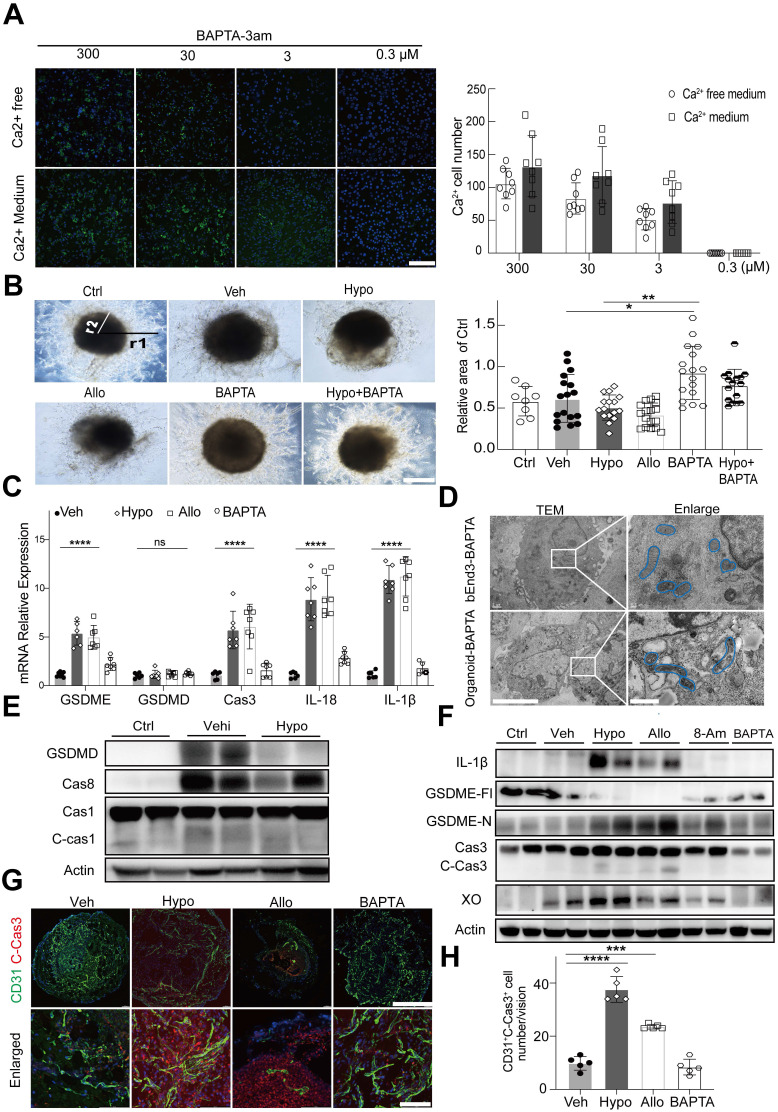
** Hypoxanthine caused GSDME-dependent pyroptosis of endothelial cells by inducing intracellular Ca^2+^ accumulation.** (**A**) Representative images showing the intracellular Ca^2+^ signal in bEnd.3 cells treated with different concentrations of BAPTA in medium containing Ca^2+^ or Ca^2+^-free medium at 24 h after OGD. Bar graph showing the number of Ca^2+^ positive cells at 24 h after OGD in the presence of different concentrations of BAPTA. N = 8 per group. Scale bar, 100 μm. (**B**) Representative images showing human blood vessel organoids treated with vehicle, hypoxanthine, allopurinol, BAPTA and hypoxanthine+BAPTA at 24 h after OGD. Human blood vessel organoids cultured without reagents and OGD were defined as the control group. Bar graph showing the relative area of the control group at 24 h after OGD. N = 8~17 per group. Scale bar, 1 mm. (**C**) Real-time PCR was used to measure the levels of GSDME, GSDMD, caspase 3, IL-18, and IL-1β in the brains of stroke model mice after vehicle, hypoxanthine, allopurinol or BAPTA treatment. N = 7 per group. (**D**) TEM images showing the morphology of bEnd.3 endothelial cells or endothelial cells in human blood vessel organoids after treatment with BAPTA at 24 h after OGD; the mitochondrial microstructure is indicated by blue borders. Scale bar: left, 5 μm; right, 1 μm. (**E**) Western blot showing the expression of GSDMD, caspase 8, caspase 1 and cleaved caspase 1 in the brains of control mice (sham) and stroke mice treated with vehicle or hypoxanthine. (**F**) Western blot showing the expression of IL-1β, full length GSDME (GSDME-Fl), GSDME-N, XO, and cleaved caspase 3 in the brains of control (sham) mice and stroke mice treated with vehicle, hypoxanthine, allopurinol, 8-aminoinosine or BAPTA. (**G**) Immunostaining images showing cleaved caspase 3 expression in CD31+ endothelial cells from human blood vessel organoids treated with vehicle, hypoxanthine, allopurinol or BAPTA. Scale bar: top, 500 μm; bottom, 50 μm. (**H**) Bar graph showing the number of cleaved-caspase 3^+^/CD31^+^ endothelial cells in each field of view. N = 5 per group. **A** Two-way ANOVA with Tukey's multiple comparisons test. B, C, H One-way ANOVA with Dunnett's multiple comparisons test. The data are the mean ± SD. *: *p* < 0.05, **: *p* < 0.01, ***: *p* < 0.001, ****: *p* < 0.0001, vs. the control group.

Interestingly, we found that the BAPTA-treated human blood vessel organoids presented a greater vessel density and better integrity than human blood vessel organoids in the hypoxanthine group and the allopurinol group, indicating that blocking Ca^2+^ overload effectively maintained the growth of blood vessels (**Figure 6B**). TEM imaging of the microstructures of bEnd.3 cells and blood vessel organoids further revealed that BAPTA treatment improved the morphology and structure of mitochondria in endothelial cells (**Figure 6D**).

To determine whether Ca^2+^ overload mediated hypoxanthine-induced pyroptosis of endothelial cells, endothelial cells and human blood vessel organoids were treated with hypoxanthine + BAPTA. The real-time PCR data revealed that hypoxanthine and allopurinol treatment increased the expression of GSDME, caspase 3, IL-1β, and IL-18 but not GSDMD in endothelial cells and that these changes were reversed by BAPTA treatment (**Figure 6C**). Western blot analysis also revealed that, compared with vehicle, hypoxanthine did not increase the expression of caspase 8, caspase 1, cleaved caspase 1 or GSDMD (**Figure 6E**). However, hypoxanthine treatment increased the levels of GSDME-N, IL-1β, and cleaved caspase 3 and decreased the level of the full-length GSDME protein (**Figure 6F**), suggesting that hypoxanthine-induced endothelial cell pyroptosis was GSDME-dependent rather than GSDMD-dependent and that BAPTA treatment significantly (p < 0.01) reduced the levels of GSDME-N, IL-1β and cleaved caspase 3. In addition, BAPTA treatment reduced the level of cleaved caspase 3, which was highly expressed in hypoxanthine-treated human blood vessel organoids (**Figure 6G, 6H**).

## Discussion

In this study, we used SERS and LC‒MS to measure the levels of serum metabolites, identifying potential biomarkers for stroke diagnosis and prognosis prediction. We detected an increase in the activity of the "adenosine-hypoxanthine-uric acid" purine metabolic pathway, particularly an increase in hypoxanthine levels in mouse serum at 6 hours after tMCAO. Hypoxanthine has previously been proposed as a potential biomarker for brain injury, i.e., brain edema 42, and it has been reported that general anesthetic agents can cause the accumulation of purine nucleosides 43. Our findings support the notion that elevated hypoxanthine levels may serve as a risk indicator for ischemic stroke. Given the limited research on the function and mechanism of hypoxanthine, we chose to further investigate the biological and pathological roles of this metabolite. Hypoxanthine was found to increase the infarct area and exacerbate BBB disruption and behavioral deficits in mice after tMCAO. Notably, we observed a positive correlation between hypoxanthine levels and stroke severity in one of the major stroke subtypes (i.e., intracranial atherosclerosis). Mechanistically, hypoxanthine led to GSDME-dependent, but not GSDMD-dependent, pyroptosis of endothelial cells by inducing intracellular Ca2+ overload.

Cerebral ischemia causes both local and systemic metabolic disturbances. Investigating these metabolic changes allows us to determine whether certain metabolites can serve as circulating biomarkers for assessing stroke risk, diagnosing stroke and identifying stroke subtypes. Currently, metabolomics profiling is most commonly conducted via nuclear magnetic resonance (NMR) spectroscopy 44 and mass spectrometry (MS) 3. However, these state-of-the-art techniques have their own advantages and disadvantages. NMR spectroscopy requires minimal sample preparation and offers high reproducibility but has limited sensitivity, particularly for detecting metabolites present at low concentrations 44. MS, on the other hand, is more sensitive and capable of detecting a wide range of metabolites but is prone to yielding variable results, has restricted usage scenarios and high cost, and requires complex data analysis 45-47. In addition, inconsistencies in the metabolites identified by different technologies have been observed, highlighting the need for new techniques that offer high sensitivity at a lower cost. Owing to its intrinsic advantages of the ability to be used for molecular fingerprinting, ultrahigh sensitivity, and multiplex capability, label-free SERS was employed to rapidly and accurately identify differentially abundant metabolites in serum 48. This method can complement traditional MS/NMR methods for metabolomics profiling.

SERS is an optical fingerprinting technique that can be used to identify molecular species on the basis of their vibrational spectral signatures. Ultrahigh sensitivity can now be achieved in EM hotspots on metallic colloidal surfaces, allowing detection down to the single-molecule level when silver (Ag) nanocolloids are used 49-51. Label-free SERS, with the intrinsic advantages of ultrahigh sensitivity and multiplexity, has been used in numerous studies involving nontargeted molecular profiling of various biofluids (e.g., serum, urine, saliva, and cell lysates) 33, 34, 52. However, a significant challenge remains: the lack of follow-up mechanistic studies to validate potential biomarkers identified through metabolomic research. This issue is not unique to SERS but is a common obstacle in many studies involving biomarker identification 53.

In our SERS study, serum samples were subjected to appropriate pretreatments, such as ultrafiltration, prior to analysis by SERS. This step is crucial to prevent macromolecules from obstructing the interaction between metabolites and colloids 54-56. This step was overlooked in many previous studies, leading to reduced metabolite sensitivity and an inability to distinguish metabolite signals from other biocomponents, such as proteins and nucleic acids, in complex biomatrices 57, 58. To validate the differentially abundant metabolites identified from the significantly varied SERS signatures (p < 0.001 after Bonferroni correction), we further utilized conventional MS techniques for robust biomarker screening. The SERS serum metabolic profiles were then used to develop an SVM-based classification model, which was able to discriminate samples with an AUC as high as 97%, demonstrating the potential for accurate and rapid diagnosis with minimal invasiveness. SERS-based serum metabolic profiling is a fast, reliable, and accurate approach for biomarker screening and diagnosis, making it broadly applicable, particularly in scenarios requiring a fast measurement speed and widespread use.

ATP, NAD^+^, and nucleic acids are abundant resources of purines that, in addition to playing critical intracellular roles, also function extracellularly as danger signals in response to cell lysis, death, degranulation, or membrane pore formation 59. ATP is metabolized to adenosine, which serves as a substrate for inosine, ultimately leading to the formation of hypoxanthine. There is substantial evidence indicating the limited production of guanine, hypoxanthine, xanthine, and uric acid, as well as their poor incorporation into mammalian nucleic acids such as DNA and RNA 60. The production of hypoxanthine is considered energy-inefficient, as it is difficult to recycle hypoxanthine back into ATP, suggesting that hypoxanthine is an ATP-wasting and redundant metabolite that needs to be eliminated. In the purine metabolism pathway, hypoxanthine is catalyzed to xanthine and subsequently to uric acid by XO. Allopurinol, an XO inhibitor, has been shown to decrease uric acid levels and oxidative stress, thereby improving stroke outcomes 61, 62. However, clinical studies have shown that high doses of allopurinol can cause side effects. Inosine, as a precursor of hypoxanthine, plays a crucial role in converting hypoxanthine back to inosine, thus preventing XO from converting hypoxanthine to uric acid. This process is vital for treating stroke and purine recycling. The controversy lies in whether hypoxanthine itself or the final product, uric acid, exacerbates brain injury, as uric acid is associated with an increased risk of cardiovascular disease 63. This catabolic pathway produces reactive oxygen species, which are harmful in the context of ischemic stroke.

The direct effects of hypoxanthine may depend on the presence of uric acid in the body. However, completely preventing uric acid formation in mammals is currently impossible, highlighting the need for further investigation. We speculate that allopurinol, by inhibiting XO, leads to the accumulation of hypoxanthine. The similarities observed between the allopurinol-treated group and the hypoxanthine group could be attributed to the secondary effect of excess hypoxanthine. In addition, it is possible that allopurinol is toxic and contributes to endothelial cell death. Our findings provide valuable insights into the potential side effects of allopurinol. However, we did not clarify the underlying mechanisms of the toxicity observed in the allopurinol group or the ability of allopurinol to induce endothelial cell death, which underscores the need for further investigations in this area.

Previous studies on hypoxanthine have focused primarily on its relationship with the progression of stroke. Hällgren *et al.* reported that CSF-derived hypoxanthine is upregulated in patients with acute cerebrovascular lesions (CVLs) and global cerebral ischemia (GCI), and is significantly correlated with the maximum lesion volume 11. Heiss and colleagues demonstrated that a progressive increase in the concentration of hypoxanthine is associated with an increased infarct volume 12. Recently, Irvine *et al.* proposed that hypoxanthine can serve as a biomarker of brain edema and as an indicator of the treatment response to glibenclamide in stroke patients 41. These studies suggest that hypoxanthine may play harmful roles in stroke, but its underlying mechanism has never been studied. In our study, we demonstrated that hypoxanthine induces GSDME-dependent pyroptosis in endothelial cells directly rather than through GSDMD activation, providing deeper insight into why hypoxanthine plays a harmful role after stroke. Using human blood vessel organoids, a stroke mouse model, and clinical patient samples, we demonstrated that hypoxanthine-induced BBB breakdown is mediated by GSDME-dependent pyroptosis in endothelial cells, which is associated with Ca2+ overload. This was evidenced by the fact that chelating intracellular calcium ions with BAPTA-AM reduced endothelial cell pyroptosis. Moreover, we showed that SERS, a novel technique we previously developed 34, has broad implications, potentially facilitating the diagnosis of a wide range of metabolic diseases other than stroke. In our study, we identified hypoxanthine as an important metabolite for mediating GSDME-dependent pyroptosis of endothelial cells during stroke, which is associated with Ca^2+^ overload. Our study provides new targets for developing strategies to protect the BBB after stroke.

## Supplementary Material

Supplementary figures and tables.
