# Supplementary material for: Hypoxanthine is a metabolic biomarker for inducing GSDME-dependent pyroptosis of endothelial cells during ischemic stroke
Source: Theranostics. 2024 Sep 16;14(15):6071–87. doi: 10.7150/thno.100090 (PMC11426240; doi:10.7150/thno.100090)
Supplement: Supplementary file 1 — Supplementary figures and tables. [file thnov14p6071s1.pdf]

Supplementary Information

Hypoxanthine is a metabolic biomarker for inducing GSDME-dependent pyroptosis of endothelial cells during ischemic stroke

Jing Ye[1#], Xinyuan Bi[1#], Shiyu Deng[1], Xianghui Wang[1], Ze Liu[1], Qian Suo[1], Jiao Wu[1,2], Haoran Chen[1], Yong Wang[3], Kun Qian[1,2], Rubing Shi[1], Jing Zhao[4*], Guo-Yuan Yang[1*], Jian Ye[1,2,5*], Yaohui Tang[1*]

*Corresponding author. Email

yaohuitang@sjtu.edu.cn (Yaohui Tang);

yejian78@sjtu.edu.cn (Jian Ye)

gyyang@sjtu.edu.cn (Guo-Yuan Yang)

zhao_jing@fudan.edu.cn

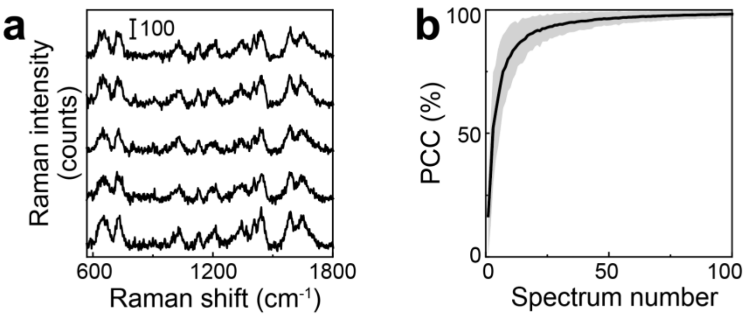

Figure S1. Estimation of spectrum number per measurement for robust SERS profiling. (a) Signal fluctuation across successively acquired spectra in one measurement. (b) Pearson's correlation coefficient (PCC) versus the spectrum number per measurement. The black line for the mean value in all samples and the gray shade for the standard deviation (n = 120).

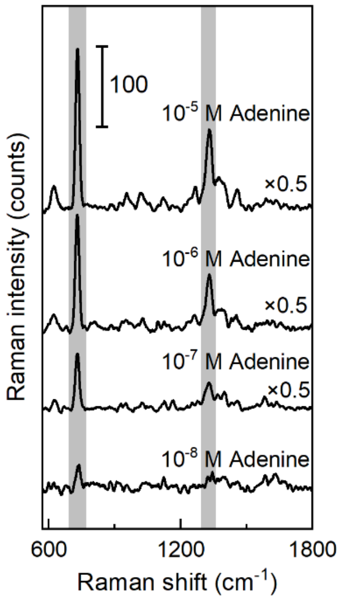

Figure S2. SERS spectra of adenine at different concentrations. The intensity of the characteristic peak of adenine as indicated by the grey shade is positively correlated with the concentration, indicating the capability of quantification even down to 10^{-8} M. While the peak position remains constant, validating the biomarker identification based on the characteristic peaks.

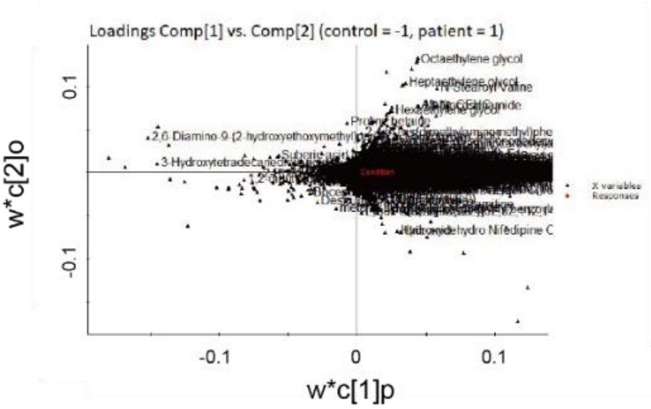

Figure S3. Permutation test of OPLS-DA in Figure 2k. The permutation plot displays the correlation coefficient between the original y variable and the permuted y variable on x axis versus the cumulative on the y axis, indicating the effective prediction ability.

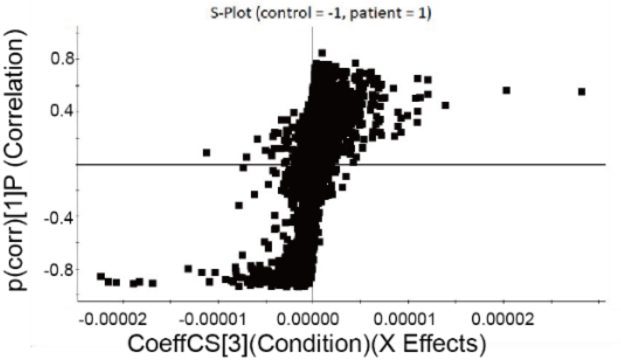

Figure S4. Metabolite detection of human serum by the conventional HPLC-MS-MS in positive ion mode. S-plots of the ischemic stroke group.

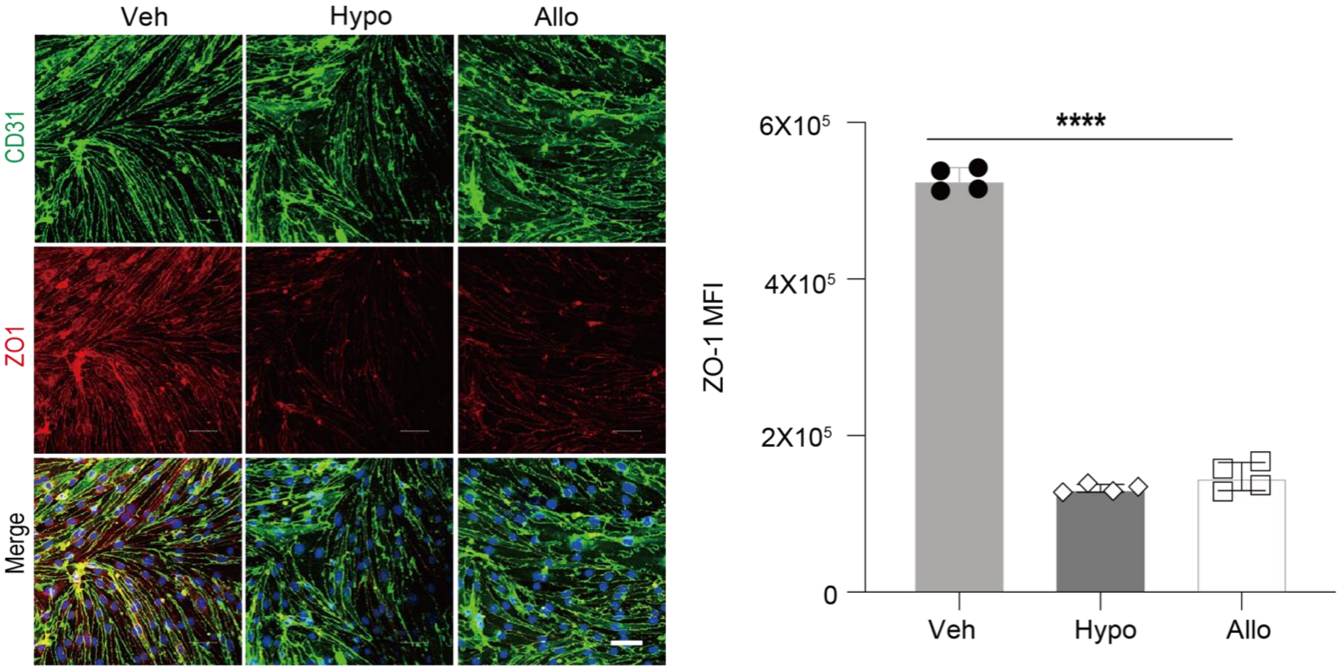

Figure S5. Hypoxanthine treatment reduced the expression of tight junction protein ZO-1 in endothelial cells. Immunostaining and quantification of tight junction protein ZO-1 expression in endothelial cells treated with vehicle (Veh), hypoxanthine (Hypo) or allopurinol (Allo). Scale bar, 100 µm. One-way ANOVA with Dunnett's multiple comparisons test. N = 4. Data are mean ± SD. ****: $p < 0.0001$, vs vehicle.

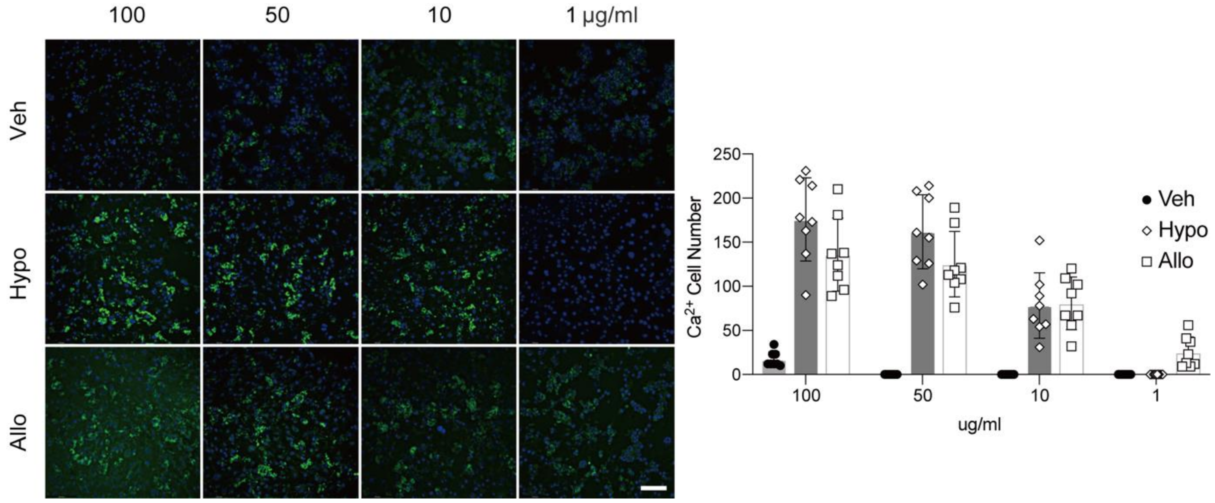

Figure S6. Hypoxanthine and allopurinol induced intracellular Ca^{2+} accumulation in endothelial cells in a dose-dependent manner. Representative images of Ca^{2+} signal in bEnd.3 cells treated with different concentrations of vehicle, hypoxanthine, or allopurinol at 24 h after OGD. Scale bar, 100 µm. Two-way ANOVA with Bonferroni multiple comparisons test. N = 8. Data are mean ± SD.

Table S1. Primer sequence.

β-actin-F	5’-GGCTGTATTCCCCTCCATCG-3’
β-actin-R	5’-CCAGTTGGTAACAATGCCATGT-3’
IL-1β-F	5’-AGAGCCCATCCTCTGTGACT-3’
IL-1β-R	5’-GCTCATATGGGTCCGACAGC-3’
IL-18-F	5’-GACTCTTGCGTCAACTTCAAGG-3’
IL-18-R	5’-CAGGCTGTCTTTTGTCAACG-3’
caspase 3-F	5’-CTCTGGTTTTCGGTGGGTGT-3’
caspase 3-R	5’-CTTCCATGTATGATCTTTGGTTCC-3’
GSDMD-F	5’-TCTGCCCTCAACACTTCTGG-3’
GSDMD-R	5’-TGCAGCCACAAATAACTCAGC-3’
GSDME-F	5’-TGCAACTTCTAAGTCTGGTGACC-3’
GSDME-R	5’-CTCCACAACCACTGGACTGAG-3’

Table S2. Ischemic stroke patients information

**Characteristic**	**Stroke (n=51)**	**Control (n=69)**	**P value**
Age, year median ± SD	74 ±11.309	50 ± 8.217	0.3192
Sex, male, n (%)	Male, 26 (51)	Male, 40 (58)	0.8973
**TOAST, n (%)**	LAA, 30(55.6)<br>CE, 15 (27.8)<br>SAO, 8 (14.8)	Ns	Ns
**Therapy**	tPA, 29 (57)<br>Thrombectomy, 22 (42)	Ns	Ns
**Medical history**			
Previous stroke, n (%)	6 (11)	Ns	Ns
Hypertension, n (%)	38 (75)	30（43）	0.3506
Diabetes Mellitus, n (%)	16 (31)	25（36）	0.8156
Arial fibrillation, n (%)	12 (23)	22（22）	0.5001
**On admission**			
NIHSS score, median (SD)	9 (5.69)	Ns	Ns

SD (Standard Deviation); TOAST (Trial of Org 10172 in Acute Stroke Treatment); LAA (large-artery atherosclerosis); CE (cardio embolism); SAO (small artery occlusion); SUE (stroke of undemonstrated etiology); tPA (Tissue plasminogen activator); NIHSS (National Institute of Health Stroke scale).

Table S3. Supplemental materials

REAGENT or RESOURCE	SOURCE	IDENTIFIER
**Antibodies**		
Anti- Caspase 3 antibody	Cell Signaling Technology	Cat#9662
Anti- Cleaved Caspase-3 (Asp175) antibody	Cell Signaling Technology	Cat#9661
Anti-cleaved GSDME N-terminal	Abcam	Cat# ab222407
Anti- DFNA5/GSDME Ab antibody	Affinity Biosciences	Cat#AF4016
Anti- IL-1 B/IL1B Ab (B122)antibody	Santa Cruz	Cat#sc-12742
Anti-DFNA5/GSDME Ab antibody	Abcam	Cat#a ab21591
Anti- GSDMDC1 AB (64-Y) antibody	Santa Cruz	Cat# sc-81868
Anti- Caspase-8 (1.1.40) antibody	Santa Cruz	Cat# sc-81656
Anti- Caspase-1 antibody	Santa Cruz	Cat# sc-56036
Anti- Caspase 1 rabbit pAb	Zenbio	Cat#342947
Anti- Cleaved-Caspase 1 rabbit pAb	Zenbio	Cat#341030
Anti- Cleaved-Caspase 3 rabbit pAb	Zenbio	Cat#R23727
Anti- Xanthine Oxidase antibody	Santa Cruz	Cat# sc-398548
Anti-Xanthine Oxidase [EPR4605]	Abcam	Cat#ab109235
Anti-ZO-1antibody	Invitrogen	Cat#61-7300
Anti- CD31 antibody	R&D	Cat#AF3628
Anti-CD31 antibody	R&D	Cat#AF806
horseradish peroxidase-conjugated goat anti-rabbit IgG	HUABIO	Cat#HA1001
horseradish peroxidase-conjugated goat anti-mouse IgG	HUABIO	Cat#HA1006
horseradish peroxidase-conjugated rabbit anti-goat IgG	HUABIO	Cat#HA1005
Second antibodies donkey anti-mouse alexa fluor 594	Invitrogen	Cat#A21203
Second antibodies donkey anti-goat alexa fluor 647	Invitrogen	Cat#A21447
**Chemicals, peptides, and recombinant proteins**		
DMEM/F12	Gibco	Cat#11330-032
DMEM Ca2+ free medium	Gibco	Cat#21068028
Hypoxanthine	Sigma-Aldrich	Cat#H9337
Citrate trisodium (98%)	Aladdin	Cat#S189183
Silver nitrate (AR, 99.8%)	Aladdin	Cat# S116264

Cysteamine (95%)	Aladdin	Cat# C106461
Phenylalanine (HPLC, > 98.0%)	Aladdin	Cat# P103489
Hypoxanthine (99%)	Aladdin	Cat# H108384
Uric acid (99%)	Aladdin	Cat# U105582
Riboflavin (99.55%)	MedChemExpress	Cat#HY-B0456
Adenine (HPLC, ≥ 99.5%)	Macklin	Cat# A800685
Nicotinic acid (≥ 99%)	Yuanye	S13023
Mass spectrometry metabolite library	Sigma-Aldrich	MSMLS
8-Aminoguanine	Sigma-Aldrich	Cat#SML1856
Allopurinol	Sigma-Aldrich	Cat#A8003
JC-1(Synonyms:CBIC2)	MedChemExpress	Cat#HY-15534
Fluo-3AM	MedChemExpress	Cat#HY-D0716
penicillin/streptomycin	Meilunbio	Cat#MA0110-1
Accutase	STEMCELL	Cat#07920
poly-l-ornithine	Sigma-Aldrich	Cat#L4544
mTeSR™ medium	STEMCELL Technologies	Cat#85850
Matrigel	Corning	Cat#356231
ReleSR	STEMCELL	Cat#100-0484
Y-27632	STEMCELL Technologies	Cat#72308
Mesodermal induction medium[APEL2]	STEMCELL	Cat#05270
CHIR99021	STEMCELL	Cat#72054
VEGF	YEASEN	Cat#91502ES10
BMP4	YEASEN	Cat#92053ES10
bFGF	YEASEN	Cat#91330ES10
EGM medium	Lonza	Cat#CC-3162
Tamoxifen	MedChemExpress	Cat# HY-13757A
Tetrazolium chloride	Sigma-Aldrich	Cat#T-8877
TRI reagent	MRC	Cat#TR118
RIPA lysis buffer	EMD Millipore	Cat#20-188
phosphatase inhibitor cocktail	ROCHE	Cat#11697498001
Proteinase inhibitor cocktail	ROCHE	
**Critical commercial assays**		
Xanthine/Hypoxanthine Assay	Abcam	Cat#ab155900
Xanthine Oxidase Activity	Abcam	Cat#ab102522
Uric Acid Assay	Abcam	Cat# ab65344
Hifair II 1st Strand cDNA Synthesis SuperMix for qPCR (gDNA digester plus)	YEASEN	Cat#11123ES60
Hieff qPCR SYBR Green master mix (High rox)	YEASEN	Cat#11203ES08
TRIzol reagent box	Thermo Fisher Scientific	Cat#15596018

**Experimental models: Organisms**		
C57BL/6J mice	Beijing Charles River	N/A
**Software and algorithms**		
Lax		
ImageJ software	ImageJ	N/A
Prism v.7 software	GraphPad	N/A
Xcalibur 3.0	Thermo fisher Scientific	N/A
Progenesis QI v 2.3	Waters	N/A
Ezinfor V3.0.3	Umetrics	N/A
Python (version 3.9.16)	Python Software Foundation	N/A
LabSpec 6 (version 6.4.4.10)	Horiba Scientific	N/A
Matlab (version R2022b)	MathWorks	N/A

Table S4. Comparison of serum metabolites between ischemic stroke patient and control

Accepted Compo	Accepted Description	Anova (p)	q Value	Max Fold Change
HMDB0001933	Furosemide	0.00048635	0.000471154	588.8124158
HMDB0255703	Non-sulfonylurea	0.00053061	0.000510471	175.4223065
HMDB0252034	Ethyl 6-chlorochroman-2-carboxylate	4.28E-05	5.14E-05	127.9387857
HMDB0029722	1,2,5-Trimethyl-1H-pyrrole	7.94E-05	9.02E-05	90.98485273
HMDB0240252	Salicyluric beta-D-glucuronide	3.91E-07	7.17E-07	75.0981604
HMDB0242638	Doramectin	5.82E-05	6.83E-05	63.20475577
HMDB0038738	Ethyl 3-hydroxyoctanoate O-[glucosyl-(1->6)-glucoside]	0.00010382	0.000115036	57.71727194
HMDB0038574	Lansiumamide A	9.30E-06	1.28E-05	57.31824833
HMDB00157	Hypoxanthine	2.16E-12	8.14E-12	51.68461924
HMDB0255805	Chloro-{methyl-[1-(2-methyl-5,6-dihydro-[1,4]oxathiin-3-yl)-methanoy	0.00079019	0.000731158	50.02769421
HMDB0247526	N(G)-Nitroarginine-4-nitroanilide	1.28E-11	4.41E-11	45.11379552
HMDB0062570	Erythro-5-hydroxy-L-lysinium(1+)	1.62E-13	6.84E-13	32.40426589
HMDB0033757	Marcanine A	1.47E-05	1.94E-05	31.3487609
HMDB0038315	28-Galloylglucosylpomolate 3-arabinoside	0.00022175	0.000232197	30.27811136
HMDB0242124	Dibutyl sulfosuccinate	2.92E-07	5.47E-07	26.71026234
HMDB0257086	N-(1-Carboxy-3-carboxanilidopropyl)alanylproline	1.61E-05	2.11E-05	16.07329022
HMDB0014905	Benzquinamide	1.30E-09	3.43E-09	15.74153395
HMDB0037153	S-2,5-Dimethyl-3-furanyl 3-methylbutanethioate	5.68E-12	2.04E-11	15.44445283
HMDB0246169	2-Mercapto-3-phenylthieno[3,2-d]pyrimidin-4(3H)-one	2.93E-05	3.62E-05	14.33613768
HMDB0011654	2-(3-Carboxy-3-(methylammonio)propyl)-L-histidine	1.19E-06	1.98E-06	13.0437765
HMDB0256350	Perphenazine decanoate	3.81E-11	1.23E-10	12.68713325
HMDB0244626	Desmethyl rizatriptan	1.49E-06	2.41E-06	11.65040594
HMDB0245935	3-Methylsulfonyl-4-piperidin-1-ylbenzoyl guanidine	1.11E-16	6.05E-16	10.78803898
HMDB0255366	2-[(3-Iodophenyl)methyl]-1-[[N'-[(3-iodophenyl)methyl]carbamimidoyl]	0.0004774	0.00046346	10.70725048
HMDB0033483	6,7-Dihydro-4-(hydroxymethyl)-2-(p-hydroxyphenethyl)-7-methyl-5H-	0.00088677	0.000810212	10.58700997
HMDB0250537	Crimidine	4.67E-13	1.89E-12	10.34897315
HMDB0010320	Cortolone-3-glucuronide	3.10E-11	1.01E-10	10.01399877
HMDB0249821	ceritinib	8.22E-07	1.42E-06	10.00085324
HMDB0244021	1,1-Dichloroheptane	5.32E-14	2.37E-13	9.855229195
HMDB0256196	Chembl4238926	5.65E-13	2.26E-12	9.697804361
HMDB0000739	Isodesmosine	9.57E-05	0.000106872	9.527567954
HMDB0255616	Niobium(V) oxide	1.76E-09	4.56E-09	8.757590741
HMDB0094704	N-Propionylmethionine	1.11E-16	6.05E-16	8.420112093
HMDB0014644	Cimetidine	1.11E-16	6.05E-16	7.917252929
HMDB0005076	13,14-Dihydro PGF-1a	7.32E-14	3.21E-13	7.872678857
HMDB0256990	pyroglutamyl-histidyl-glycine	3.71E-12	1.37E-11	7.793671825
HMDB0059977	4-Hydroxy-5-(dihydroxyphenyl)-valeric acid-O-methyl-O-sulphate	8.22E-12	2.90E-11	7.469368416
HMDB0060834	N-Acetylserotonin sulfate	1.22E-11	4.21E-11	7.281641988
HMDB0247747	Abecarnil	1.11E-12	4.31E-12	7.266604938
HMDB0029367	Sanguinarine	9.11E-09	2.17E-08	7.195765477
HMDB0247589	9-Deaza-9-(3-thienylmethyl)guanine	3.10E-11	1.01E-10	7.141102735
HMDB0249909	Galactocerebroside	2.52E-09	6.39E-09	7.100953573
HMDB0033335	Antibiotic X 14889C	4.10E-11	1.31E-10	7.00717749
HMDB0244433	Vorozole	4.74E-10	1.33E-09	6.966408545
HMDB0258388	Sorbinil	1.28E-10	3.87E-10	6.650528508
HMDB0252020	Ethoprophos	7.95E-14	3.47E-13	6.544754665
HMDB0029367	Sanguinarine	6.51E-09	1.57E-08	6.493889878
HMDB0247498	Butyryl trihexyl citrate	2.78E-10	8.00E-10	6.441786698
HMDB0247875	(Z)-7-[(1S,4R,6R)-4-[(E)-Oct-6-enyl]-2,3-diazabicyclo[2.2.1]hept-2-en-	8.00E-11	2.48E-10	6.398357155
HMDB0252347	Fluocinolone	1.48E-05	1.96E-05	6.385914174
HMDB0241595	(5Z,8Z,10E,14Z,17Z)-12-Hydroxyicosa-5,8,10,14,17-pentaenoylcarni	2.43E-05	3.06E-05	6.336730384
HMDB0259276	Tris(2-chloroethyl) phosphate	6.95E-06	9.89E-06	6.303539505
HMDB0029782	6-Epi-7-isocucurbic acid glucoside	2.33E-14	1.07E-13	6.269225917
HMDB0246374	4-Azidobenzenesulfonamide	3.33E-16	1.76E-15	6.195299824
HMDB0303432	Calcium citrate	0.00016688	0.00017848	6.174775172
HMDB0259625	2,4-Pyrimidinediamine, 5-(5-(1-piperazinylmethyl)-1,3,4-oxadiazol-2-	1.94E-14	8.98E-14	6.170533447
HMDB0246374	4-Azidobenzenesulfonamide	2.55E-11	8.48E-11	6.16886088
HMDB0248879	Batelapine	2.59E-05	3.24E-05	6.160594357
HMDB0000394	3-Hydroxytetradecanedioic acid	9.99E-16	5.14E-15	6.125766202
HMDB0259952	1-Cycloheptyl-1-(9H-fluoren-2-ylmethyl)-3-(2,4,6-trimethylphenyl)ure	9.97E-11	3.05E-10	5.990684565
HMDB0248956	Benoxaprofen	1.08E-08	2.54E-08	5.98503962
HMDB0259712	urapidil	1.11E-16	6.05E-16	5.920339535
HMDB0255529	Neosalacinol	2.23E-13	9.28E-13	5.919354842
HMDB0040824	Tsangane L 3-glucoside	1.21E-11	4.19E-11	5.817481926
HMDB0246720	5-(4-Fluorophenyl)-3-(3-nitrophenyl)-1,2,4-oxadiazole	8.58E-13	3.37E-12	5.754720603
HMDB0031885	6-Hydroxypentadecanedioic acid	1.08E-12	4.21E-12	5.727348964
HMDB0060538	Nordiazepam	1.07E-10	3.28E-10	5.657318625
HMDB0254042	Leupeptin	1.06E-11	3.70E-11	5.488533065
HMDB0258508	Estreptoquinasa	3.92E-09	9.72E-09	5.284556616
HMDB0259434	4H-Thieno(2,3-b)thiopyran-2-sulfonamide, 5,6-dihydro-6-(3-methoxy	0.00046725	0.00045471	5.218390683
HMDB0015150	Sulfamethoxazole	1.67E-08	3.83E-08	5.141039278
HMDB0256398	Phenazocine	1.68E-11	5.69E-11	5.139415331
HMDB0039947	Pisumionoside	4.87E-09	1.20E-08	5.023945225
HMDB0059931	2,3-Diacetoxypropyl stearate	1.14E-07	2.29E-07	5.011095801
HMDB0012087	SM(d18:0/18:0)	1.97E-06	3.13E-06	4.920443622
HMDB0014769	Clofarabine	9.65E-09	2.29E-08	4.86302871
HMDB0248054	N-(3-Chlorophenyl)-6,7-dimethoxyquinazolin-4-amine	2.56E-05	3.21E-05	4.796348887
HMDB0248566	1,3(2H,4H)-Isoquinolinedione, 2-(2-(4-(2-methoxyphenyl)-1-piperazi	5.80E-11	1.82E-10	4.791507273
HMDB0033757	Marcanine A	2.89E-15	1.43E-14	4.753547005
HMDB0041946	N-Nitrosoproline	1.93E-10	5.69E-10	4.65400618
HMDB0094708	Tetraethylene glycol	9.29E-09	2.20E-08	4.623660124
HMDB0059609	albendazole S-oxide	9.37E-08	1.91E-07	4.559820672
HMDB0029887	Sorbitan palmitate	2.42E-10	7.03E-10	4.528262602
HMDB0032660	(2Z,4'Z)-2-(5-Methylthio-4-penten-2-ynylidene)-1,6-dioxaspiro[4.4]no	1.11E-08	2.60E-08	4.474122729
HMDB0242753	Vertilmicin	9.86E-11	3.02E-10	4.461910364
HMDB0259640	Benzonitrile, 3-amino-4-((2-((dimethylamino)methyl)phenyl)thio)-	2.22E-16	1.19E-15	4.44949376
HMDB0247381	H-Arg(NO2)-Obzl	3.33E-16	1.76E-15	4.438178874
HMDB0303294	Sodium decylbenzenesulfonate	0.00029675	0.000303084	4.410913229
HMDB0304801	Ile-Val-Val	1.62E-07	3.17E-07	4.395228641
HMDB0304214	5-hydroxy-2-oxo-4-ureido-2,5-dihydro-1H imidazole-5-carboxylate	1.89E-15	9.52E-15	4.37227215
HMDB0254628	Methyldopate	3.45E-05	4.20E-05	4.292747842
HMDB0032740	N-Hexadecanoylpyrrolidine	0.0003834	0.000380525	4.26403845
HMDB0029367	Sanguinarine	1.65E-06	2.64E-06	4.223441177
HMDB0242731	Arteether	6.35E-10	1.75E-09	4.211254162
HMDB0032959	1-Octen-3-yl glucoside	1.45E-06	2.37E-06	4.209389482
HMDB0010320	Cortolone-3-glucuronide	2.41E-06	3.75E-06	4.157546813
HMDB0246686	2-(4-Amino-2-chlorophenyl)-2-phenylacetonitrile	8.15E-08	1.69E-07	4.109561525
HMDB0257893	D-Phe-L-Pro-L-Arg-(Chloromethyl) ketone	3.47E-06	5.25E-06	4.016594789

Table S4. Mass spectrometry identified distinct serum metabolites between ischemic stroke patient and control (ANOVA, $p < 0.05$, fold change >4, OPSLSDA mode VIP >1, coefficient of variation (CV) <30%)

Table S5. Comparison of metabolites between tMCAO and control

Accepted Compound	Accepted Description	Anova (p)	q Value	Max Fold Chang
HMDB0246184	LysoPC	2.97E-11	1.72E-10	37.84162681
HMDB00157	Hypoxanthine	8.50E-07	7.24E-07	20.13678571
HMDB0032305	N-(Heptan-4-yl)benzo[d][1,3]dioxole-5-carboxamide	4.44E-16	5.03E-15	15.16865249
HMDB0032807	Niazirin	0	0	14.81950769
HMDB0304915	2-(4-Methoxyphenyl)ethyl hydrogen sulfate	0	0	13.93617529
HMDB0060017	Pyrogallol-2-O-glucuronide	3.36E-12	2.19E-11	10.0253476
HMDB0240444	3-Methylgallic acid 5-sulfate	0	0	8.736265284
HMDB0252413	Fluroxene	0	0	7.547056851
HMDB06317	trans-Hexadec-2-enoyl carnitine	0	0	7.335896696
HMDB05066	Tetradecanoylcarnitine	0	0	7.294610977
HMDB02302	3-Indolepropionic acid	3.65E-10	1.82E-09	6.932168296
HMDB05066	Tetradecanoylcarnitine	0	0	6.750587765
HMDB02005	Methionine sulfoxide	0	0	6.373197406
HMDB02014	cis-5-Tetradecenoylcarnitine	0	0	6.054708103
HMDB0244869	1H-1,2,4-Triazole-3-carboxamide	0	0	6.019038576
HMDB00696	L-Methionine	0	0	5.792575532
HMDB01830	Progesterone	0	0	5.789676533
HMDB02183	Docosahexaenoic acid	0	0	5.734095832
HMDB00222	L-Palmitoylcarnitine	0	0	5.520786596
HMDB0032369	L-Menthyl acetoacetate	0	0	5.176578666
HMDB03312	Daidzein	1.81E-05	4.74E-05	4.831319683
HMDB00222	L-Palmitoylcarnitine	0	0	4.817864744
HMDB00651	Decanoylcarnitine	9.77E-14	7.90E-13	3.993656393
HMDB00549	Gamma-Butyrolactone	0	0	3.931542412
HMDB00824	Propionylcarnitine	5.55E-16	6.17E-15	3.858051084
HMDB00532	Acetylglycine	0	0	3.826942408
HMDB03312	Daidzein	1.81E-09	8.20E-09	3.661825007
HMDB0242752	6-Methylthioinosine	0.013426	0.017192	3.555867934
HMDB04620	N-a-Acetyl-L-arginine	0	0	3.476938929
HMDB0033679	Pelargonidin 3-sophoroside	0.003255	0.005078	3.414373717
HMDB11628	Glycyrrhetinic acid	0.030999	0.034582	3.321408117
HMDB0247731	Pentaleno(1,6a-c)pyran-5-carboxylic acid, 1-(chloromethyl)-1,2,4,4a,6a,7-hexahydro-1-hydroxy-7,8-dimeth	0	0	3.250240432
HMDB01358	Retinal	0	0	2.920665612
HMDB0248182	Aloxistatin	0	0	2.855392917
HMDB0011155	PA(P-16:0/18:2(9Z,12Z))	3.71E-09	1.62E-08	2.763217454
HMDB02088	N-Oleoylethanolamine	0	0	2.760627419
HMDB00162	L-Proline	0	0	2.721266639
HMDB05045	15(S)-Hydroxyeicosatrienoic acid	0	0	2.699661526
HMDB00630	Cytosine	0	0	2.577363033
HMDB06406	Ecgonine methyl ester	1.11E-16	1.36E-15	2.573102315
HMDB00848	Stearoylcarnitine	0	0	2.463812696
HMDB00526	5a-Tetrahydrocortisol	1.19E-14	1.09E-13	2.444152079
HMDB0254486	Mesulergine	0	0	2.380445216
HMDB00014	Deoxycytidine	9.82E-12	6.02E-11	2.331847291
HMDB00721	Glycylproline	0	0	2.330325402
HMDB00210	Pantothenic acid	0	0	2.282453164
HMDB03464	4-Guanidinobutanoic acid	1.07E-09	4.99E-09	2.207425943
HMDB00273	Thymidine	5.75E-11	3.19E-10	2.165495926
HMDB02183	Docosahexaenoic acid	3.19E-14	2.71E-13	2.140799218
HMDB0245977	3-Propyl-1H-pyrazole	2.23E-05	5.78E-05	2.110894024
HMDB10391	LysoPC(20:1(11Z))	3.33E-16	3.87E-15	2.100295069
HMDB0032369	L-Menthyl acetoacetate	3.33E-16	3.87E-15	2.095794455
HMDB00205	Phenylpyruvic acid	0	0	2.03813274
HMDB11718	4-Hydroxybenzaldehyde	0	0	2.014165724
HMDB00089	Cytidine	4.64E-14	3.91E-13	1.989578973
HMDB0257361	RUCAPARIB	1.33E-13	1.06E-12	1.989057877
HMDB00036	Taurocholic acid	0.000244	0.000517	1.92639328
HMDB0257242	Rislenemdaz	0	0	1.908893558
HMDB03357	N-Acetylornithine	0	0	1.905533393
HMDB04827	Proline betaine	1.33E-10	7.03E-10	1.905380572
HMDB0028887	Histidylhistidine	1.39E-13	1.10E-12	1.868926681
HMDB03337	Oxidized glutathione	0.013558	0.017313	1.849489643
HMDB0242455	(2-((3,5-Di-t-butyl-4-hydroxyphenyl)thio)-1-methylpropoxy)acetic acid	2.80E-09	1.25E-08	1.783920521
HMDB00630	Cytosine	7.33E-15	6.85E-14	1.783012352
HMDB0303202	Calcium oleate	5.94E-12	3.76E-11	1.765939304
HMDB10390	LysoPC(20:0)	1.45E-11	8.74E-11	1.731046553
HMDB10392	LysoPC(20:2(11Z,14Z))	1.52E-08	6.04E-08	1.730144617
HMDB10399	LysoPC(22:1(13Z))	2.88E-06	8.51E-06	1.710024451
HMDB0258894	Tetraglyme	0.000585	0.001126	1.688142339
HMDB0247995	Androstane-3,17-diol 17-sulfate	1.16E-09	5.39E-09	1.673623638
HMDB0248697	Atevirdine	1.26E-09	5.83E-09	1.663703726
HMDB0257361	RUCAPARIB	7.00E-09	2.93E-08	1.601790448
HMDB10407	LysoPC(P-16:0)	9.76E-10	4.59E-09	1.601619737
HMDB0240598	LysoPE(P-18:0/0:0)	8.71E-08	3.18E-07	1.600137868
HMDB00630	Cytosine	2.70E-12	1.78E-11	1.595443544
HMDB0256571	Pipequaline	7.16E-10	3.42E-09	1.594029939
HMDB00517	L-Arginine	9.37E-07	2.96E-06	1.565056597
HMDB0005076	13,14-Dihydro PGF-1a	0	0	1.55599257
HMDB03229	Palmitoleic acid	4.23E-13	3.15E-12	1.527138706
HMDB00929	L-Tryptophan	8.08E-09	3.35E-08	1.523817777
HMDB11738	Gamma-Glutamylglutamine	0.000214	0.000458	1.516337559
HMDB0000734	Indoleacrylic acid	7.82E-09	3.26E-08	1.513546669
HMDB10401	LysoPC(22:4(7Z,10Z,13Z,16Z))	1.03E-05	2.82E-05	1.494166904
HMDB0032740	N-Hexadecanoylpyrrolidine	0.001781	0.003012	1.480650114
HMDB10397	LysoPC(20:5(5Z,8Z,11Z,14Z,17Z))	0.000146	0.000325	1.446103937
HMDB00688	Isovalerylcarnitine	1.29E-06	4.00E-06	1.433225589
HMDB00064	Creatine	3.94E-13	2.94E-12	1.431932095
HMDB01406	Niacinamide	0.000199	0.000428	1.429724651
HMDB0255383	2-Hexadec-7-enylicosa-8,11-dienedioic acid	8.45E-09	3.48E-08	1.374633444
HMDB0034625	Vinaginsenoside R2	1.36E-12	9.39E-12	1.34409516
HMDB00086	Glycerophosphocholine	0.016565	0.020478	1.336686582
HMDB0246853	Dhlnl	2.59E-14	2.26E-13	1.320675299
HMDB0303802	Campesterol glucoside	3.52E-07	1.20E-06	1.320211401
HMDB0243890	1-O-Hexadecyl-sn-glycero-3-phosphocholine	4.86E-05	0.000119	1.290822925
HMDB0033641	(3b,21b)-12-Oleanene-3,21,28-triol 28-[arabinosyl-(1->3)-arabinosyl-(1->3)-arabinoside]	3.87E-06	1.13E-05	1.287380051
HMDB10384	LysoPC(18:0)	2.47E-07	8.51E-07	1.286140236
HMDB0249823	Cerivastatin lactone	4.47E-09	1.94E-08	1.277002039
HMDB10384	LysoPC(18:0)	2.48E-08	9.68E-08	1.274356141
HMDB0012109	5,6-Dihydroxyprostaglandin F1a	1.38E-12	9.55E-12	1.273620911
HMDB00251	Taurine	1.03E-05	2.83E-05	1.26175498

Table S5. Mass spectrometry identified distinct serum metabolites between tMCAO mice and control (ANOVA, $p < 0.05$, fold change >1.25, OPSLSDA mode VIP >1, coefficient of variation (CV) <30%)
